# A Plant-Specific *TGS1* Homolog Influences Gametophyte Development in Sexual Tetraploid *Paspalum notatum* Ovules

**DOI:** 10.3389/fpls.2019.01566

**Published:** 2019-11-29

**Authors:** Carolina Colono, Juan Pablo A. Ortiz, Hugo R. Permingeat, Eduardo Daniel Souza Canada, Lorena A. Siena, Nicolás Spoto, Florencia Galdeano, Francisco Espinoza, Olivier Leblanc, Silvina C. Pessino

**Affiliations:** ^1^Molecular Biology Laboratory, IICAR, CONICET, Universidad Nacional de Rosario, Rosario, Argentina; ^2^Genetics Laboratory, IBONE, CONICET, Universidad Nacional del Nordeste, Corrientes, Argentina; ^3^UMR DIADE, IRD, Univ. Montpellier, Montpellier, France

**Keywords:** apomixis, apospory, methyltransferase, plant reproduction, *TGS1*

## Abstract

Aposporous apomictic plants form clonal maternal seeds by inducing the emergence of non-reduced (2n) embryo sacs in the ovule nucellus and the development of embryos by parthenogenesis. In previous work, we reported a plant-specific *TRIMETHYLGUANOSINE SYNTHASE 1* (*TGS1*) gene (*PN_TGS1*-like) showing expression levels positively correlated with sexuality rates in facultative apomictic *Paspalum notatum*. *PN_ TGS1*-like displayed contrasting *in situ* hybridization patterns in apomictic and sexual plant ovules from premeiosis to anthesis. Here we transformed sexual *P. notatum* with a *TGS1*-like antisense construction under a constitutive promoter, in order to produce lines with reduced transcript representation. Antisense plants developed prominent trichomes on the adaxial leaf surface, a trait absent from control genotypes. Reproductive development analysis revealed occasional formation of twin ovules. While control individuals typically displayed a single meiotic embryo sac per ovule, antisense lines showed 12.93–15.79% of ovules bearing extra nuclei, which can be assigned to aposporous-like embryo sacs (AES-like) or, alternatively, to gametophytes with a misguided cell fate development. Moreover, around 8.42–9.52% of ovules showed what looked like a combination of meiotic and aposporous-like sacs. Besides, 32.5% of ovules at early developmental stages displayed nucellar cells with prominent nuclei resembling apospory initials (AIs), which surrounded the megaspore mother cell (MMC) or the MMC-derived meiotic products. Two or more concurrent meiosis events were never detected, which suggest a non-reduced nature for the extra nuclei observed in the mature ovules, unless they were generated by proliferation and misguided differentiation of the legitimate meiotic products. The antisense lines produced a similar amount of viable even-sized pollen with respect to control genotypes, and formed an equivalent full seed set (∼9% of total seeds) after self-pollination. Flow cytometry analyses of caryopses derived from antisense lines revealed that all full seeds had originated from meiotic embryo sacs (i.e. by sexuality). A reduction of 25.55% in the germination percentage was detected when comparing antisense lines with controls. Our results indicate that *PN_ TGS1*-like influences ovule, gametophyte and possibly embryo development.

## Introduction

Apomixis (i.e. asexual reproduction *via* seeds), an odd trait described in 293 angiosperm genera, includes several developmental pathways affecting two key events of sexual reproduction: meiosis and gamete fusion (Nogler, 1984; Hand and Koltunow, 2014; Hojsgaard et al., 2014). In the sexual pathway, the seed contains a single embryo derived from the fertilization of a reduced female gamete (the egg cell) by a reduced male gamete (the pollen generative nucleus). In contrast, apomictic developmental routes involve variations of two generic alternative pathways, named *sporophytic apomixis* (cells originated from the ovule nucellus/integuments directly differentiate into embryos) and *gametophytic apomixis* (embryos arise parthenogenetically from egg cells within unreduced female gametophytes) (Crane, 2001). Moreover, gametophytic apomixis sub-classifies as *diplospory* or *apospory*, depending on the origin of the unreduced female gametophyte (i.e. the megaspore mother cells or nucellar companion cells, respectively). Interestingly, the molecular and cellular mechanisms and the driving evolutionary forces that shaped the opposite outcomes of sexuality and apomixis, biparental embryo vs. autonomous maternal embryo, remain largely speculative (Albertini et al., 2019; Leon-Martínez and Vielle-Calzada, 2019).

In the past two decades, apospory has become the most widely-studied apomixis mechanism. The elucidation of aposporous apomixis developmental routes requires detailed functional analyses of many candidate genes and integrative studies of their interactive networks. Genetic mapping (Akiyama et al., 2004; Pupilli et al., 2004; Stein et al., 2007), genomic sequencing (Calderini et al., 2006; Conner et al., 2008; Calderini et al., 2011) and extensive transcriptome surveys (Pessino et al., 2001; Rodrigues et al., 2003; Albertini et al., 2004; Laspina et al., 2008; Yamada-Akiyama et al., 2009; Polegri et al., 2010; Okada et al., 2013; Ortiz et al., 2017; Ortiz et al., 2019) have identified a unique non-recombinant, large hemizygous region associated with apospory (i.e. the Apomixis Controlling Region or ACR) and hundreds of expressed sequences possibly involved in the asexual reproductive pathway. Out of these, only a few were subjected to functional characterization in subtropical grasses, revealing apospory-related mechanisms, and proving able to recapitulate particular steps of the trait. Conner et al. (2015) showed in *Pennisetum squamulatum* that a member of the BABY BOOM AP2 family of transcription factors transcribed from the ACR locus, PsASGR-*BBM-like*, is active in egg cells and triggers parthenogenesis. Silencing of PsASGR-*BBML* in apomictic RNAi transgenic plants altered embryo parthenogenetic capacities, while ectopic expression in egg cells of sexual plants triggered the formation of haploid progeny (Conner et al., 2015). In addition, an ectopic male-expressed homologous to *BBM* (*BBM1*) induces egg cell parthenogenesis in rice and leads to clonal progeny when combined with the Mitosis instead of Meiosis (MiMe) mutant (Khanday et al., 2019). Siena et al. (2016) reported that the *ORC3* (*ORIGIN RECOGNITION COMPLEX* subunit 3) gene, which is required for endosperm formation in sexual species, might be regulated by a trans-silencing mechanism triggered by an ACR-located *ORC3* pseudogene in *Paspalum simplex*. The authors hypothesized that *ORC3* downregulation might allow the formation of an endosperm that deviates from the typical 2:1 maternal:paternal genome ratio of sexual angiosperms, as observed in apomictic *Paspalum* spp. (4m:1p; Quarin 1999). Moreover, Mancini et al. (2018) determined that expression of the MAP3K *PN_QGJ* is essential to *Paspalum notatum* aposporous development, since RNAi inhibition in the ovule nucellus resulted in significantly reduced rates of aposporous embryo sac formation with respect to the levels detected in wild type aposporous plants and transformation controls.

*P. notatum* is a rhizomatous grass species forming an agamic complex mainly composed of sexual diploid (2n = 2x = 20) and apomictic tetraploid (2n = 4x = 40) cytotypes (Ortiz et al., 2013). Tetraploid individuals reproduce mostly by obligate aposporous apomixis. Aposporous initials (AIs) differentiate from nucellar companion cells surrounding the megaspore mother cell (MMC). After a series of mitosis, one to several AIs form a non-reduced embryo sac each, which may sometimes coexists with a meiotic embryo sac originated by the legitimate megaspore. Aposporous sacs of the *Paspalum* type typically display an egg apparatus (one egg cell and one or two synergid cells), a central cell containing two polar nuclei, and no antipodal cells (Martínez et al., 2001). At anthesis, tetraploid apomictic *P. notatum* plants exhibit ovules bearing one or more aposporous embryo sacs, occasionally accompanied by a meiotic one. While apomictic embryos are generated by parthenogenesis from unreduced egg cells, central cell fertilization (pseudogamy) is required for endosperm development and viable seed formation, despite of an imbalanced maternal:paternal genomic ratio (4:1) (Ortiz et al., 2013). Full sexual tetraploid cytotypes were not found in nature, but several genotypes have been experimentally produced by colchicine treatment of diploid seeds or by crosses (Quarin et al., 2001; Quarin et al., 2003). They were subsequently used for genetic mapping of apomixis factors, apomictic vs. sexual differential expression analyses, and breeding (Martínez et al., 2001; Stein et al., 2004; Stein et al., 2007; Laspina et al., 2008; Acuña et al., 2011; Ortiz et al., 2017; Zilli et al., 2018).

Comparative transcriptome analysis involving florets of apomictic and sexual *P. notatum* plants led us to identify hundreds of candidate genes with differential expression patterns (Laspina et al., 2008; Ortiz et al., 2017). In particular, we detected a plant-specific RNA methyltransferase that belongs to the TRIMETHYGUANOSINE SYNTHASE 1 family (*PN_TGS1*-like), whose representation was significantly reduced in reproductive organs of apomictic genotypes compared to sexual genotypes (Laspina et al., 2008; Siena et al., 2014). qPCR analyses revealed that *PN_TGS1*-like expression peaked at anthesis and positively correlated with the percentage of ovules displaying meiotic sacs (Siena et al., 2014). *In situ* hybridization experiments further showed *PN_TGS1*-like upregulation in the nucellus of sexual plant ovules throughout development, from premeiosis to anthesis (Siena et al., 2014). Altogether, these observations led us to hypothesize that *PN_TGS1*-like might be involved in the repression of AI formation in the nucellus (Siena et al., 2014).

In yeast and mammalian, PRIP-interacting trimethylguanosine synthase (*TGS1*) plays a dual role as ERK2-controlled transcriptional coactivator and effector of non-coding RNAs m(7)G cap conversion into m(2,2,7)G, a process involved in numerous cellular functions, such as mRNA splicing, telomere length maintenance and cell cycle progression (Zhu et al., 2001; Misra et al., 2002; Mouaikel et al., 2002; Franke et al., 2008; Qiu et al., 2011; Kapadia et al., 2013; Martínez et al., 2017). In yeast, mammalian, and *Drosophila* genomes, TGS1 contains a single RNA-cap guanine-N2 methyltransferase domain, and is encoded by a unique gene. Conversely, plant genomes contain at least one additional gene encoding the RNA methyltransferase domain and a long, extended N-terminal region, containing a WW interaction domain (Siena et al., 2014). The specific biological function of this plant-specific variant has not been determined yet, however it was recently associated with chilling tolerance in *Arabidopsis* (Gao et al., 2017). The objective of this work was to investigate the effects of a *PN_TGS1*-like repression during sexual reproductive development and, particularly, its possible role in controlling cell fate identity within the ovule, as previously posed by Siena et al. (2014). To achieve this, we produced RNAi transformants from sexual *P. notatum* plants, which showed attenuated expression levels for *PN_TGS1*-like during female reproductive development. Herein, we provide a detailed description of the reproductive behavior of these RNAi lines. Our results indicate that *PN_TGS1*-like downregulation alters the normal pattern of gametophyte formation, pointing to a major role in cell growth and differentiation within plant ovules.

## Materials and Methods

### Plant Material

The *P. notatum* material used in this work consisted of tetraploid plants of different origins: Q4117, a highly apomictic natural accession of Southern Brazil (Ortiz et al., 1997); Q4188, a sexual genotype obtained experimentally (Quarin et al., 2003); three sexual individuals (JS-36, JS-58 and JS-83) and three apomictic individuals (JS-9, JS-71 and JS-130) from an F_1_ segregating population originated from the cross between Q4188 and Q4117 (Stein et al., 2004); a fully sexual synthetic tetraploid (2n = 4x = 40) population (SSTP) of *P. notatum* obtained by Zilli et al. (2015, 2018). Genotypes Q4188 and Q4117, as well as the SSTP population, belong to the living *Paspalum* germplasm collection established at IBONE, CONICET-UNNE, Corrientes, Argentina. All materials were established as experimental plots at IICAR, CONICET-UNR, Rosario, Argentina.

### Plant Transformation

To build the *PN_TGS1*-like silencing vector (pAct1-F1as) we started from plasmid pAct1-gfbsd2 (Ochiai-Fukuda et al., 2006), which carries an enhanced green fluorescent protein gene (*EGFP*) cloned downstream of the rice Actin1 promoter. The *EGFP* sequence was replaced by F1, a 733-bp *PN_TGS1*-like antisense fragment flanked by positions 7-739 within the *PN_TGS1* sequence, GeneBank accession KM114905 (Siena et al., 2014) (**Supplementary Figure 1**). F1 comprises the last 63 nt of the *PN_TGS1*-like 5’UTR region and the first 670 nt of the *PN_TGS1*-like CDS, excluding the conserved RNA methyltransferase domain. F1 was originally cloned into the pGEM-TEasy vector (PROMEGA Madison, WI, USA). For construction, plasmids pAct1-gfbsd2 and pGEM-TEasy-F1 were digested with *Spe*I and *Not*I restriction enzymes, and the bands corresponding to F1 (733 bp) and pAct1-gfbsd2 (5510 bp) were purified by electroelution after electrophoresis (Sambrook and Russell, 2001). The F1 fragment and the digested pAct1-gfbsd2 plasmid were then ligated with DNA T4 ligase (PROMEGA) and the resulting construct, pAct1-F1as, was amplified by transforming DH5α competent cells. Finally, pAct1-F1as was sequenced at Macrogen Inc. (Seoul, Korea) to check position, sense and integrity of the F1 fragment. Embryogenic calli were produced from mature seeds of the sexual synthetic tetraploid SSTP population obtained by Zilli et al. (2018) (see above) following the tissue culture procedures in MM5 medium described by Mancini et al. (2014). Transformation experiments were carried out using a mixture of pAct1-F1as and pGFPBAR (the latter carries the GFP reporter gene and the selectable BAR gene cloned under the 35S cauliflower mosaic virus and the maize ubiquitin promoters, respectively) (Huber et al., 2002). A gene gun device (BIOMICS, Brasilia, Brazil) was used for particle delivery, at a compressed helium pressure of 900 psi and a microprojectile flight distance of 5 cm (Mancini et al., 2014). Before and after bombardment, calli were treated with osmotic medium, as indicated in Mancini et al. (2014). Bombarded calli were transferred to selection medium containing 1 mg/L ammonium glufosinate for 4 weeks (with sub-cultures every 15 days) (Mancini et al., 2014). Then, resistant calli were transferred to shoot induction media MRV1 and incubated at 28°C in a culture chamber with a photoperiod of 14-h light/d, as indicated in Mancini et al. (2014). After 40 days, calli with shoots longer than 5 cm were transferred to flasks containing rooting medium MEEV3 (Mancini et al., 2014) and incubated at 28°C in a culture chamber with a photoperiod of 14-h light/d. Finally, seedlings showing good development were washed with distilled water to eliminate remnants of culture media. Regenerated plants from non-transformed calli (R0) and bombarded, selected calli (E1/E2) were transferred to pots containing 1:1 soil/vermiculite and cultured in a GMO confined growth chamber until maturity. At flowering, inflorescences were bagged for allowing self-pollination.

### Identification of Positive Antisense Lines

To analyse the presence of the pAct1-F1as construction, genomic DNA extracted with a modified CTAB method (Paterson et al., 1993) was subjected to nested-PCR to specifically amplify a 530-bp F1 region (see **Supplementary Table 1** for primers): first, a 1270-bp fragment containing the F1fragment was amplified using Act-Nos2-Upper and -Lower primers and, then, used as template in nested PCR to generate a 530-bp product using the Act-Nos2-Upper primer in combination with Nested Lower primer. In addition, a fragment of the *Agrobacterium* NOS-T terminator sequence, present in both pAct1-F1as and pGFPBAR was tested using NOS-Upper and -Lower primers. Amplification were carried out in 25 µl final volume reactions containing 1×Taq polymerase buffer (INBIO HIGHWAY, Tandil, Argentina), 1.5 mM MgCl_2_, 0.2 mM dNTPs, 0.5 µM each primer, 1.25 U Taq polymerase (INBIO HIGHWAY) and 50 ng genomic DNA or 1 µl of 1/1,000 to 1/10,000 dilution of the first amplification. PCR cycles consisted of an initial denaturation step at 94°C for 5 min, followed by 35 cycles of 30 s at 94°C, 1 min at the corresponding Ta (temperature of annealing, listed in **Supplementary Table 1**), 25 s to 1.5 min at 72°C, depending on the size of the fragment to be amplified, and a final extension of 10 min at 72°C.

### Expression Level Quantitation

Total RNA was extracted from leaves and flowers (at anthesis) using the SV Total RNA Isolation Kit (PROMEGA) and reverse transcribed with Superscript II (INVITROGEN, Carlsbad, CA, USA) following manufacturer recommendations. Quantitative PCR reactions (final volume: 20 µl) included 0.5 µM gene-specific primers, 1X Real Mix qPCR (BIODYNAMICS, Buenos Aires, Argentina) and 20 ng of cDNA. In each experiment, two biological replicates were processed, each one including three technical replicates and a negative control (PCR reaction with no template). Amplifications were performed in a Rotor-Gene Q thermocycler (QIAGEN, Hilden, Germany), as follows: 2 min at 94°C followed by 45 cycles of 94°C for 15 s, 57°C for 30 s, and 72°C for 17 s, and a final elongation step of 5 min at 72°C. The *PN_TGS1-like* specific primers were qPCR-Upper/Lower (**Supplementary Table 1**). Relative quantitative expression levels of *PN_TGS1*-like were assessed using the REST-RG 2009 software (QIAGEN) with *β-TUBULIN* as internal reference, as recommended by Felitti et al. (2011); Ochogavía et al. (2011) and Podio et al. (2014a) for analogous comparative systems. All primers used are shown in **Supplementary Table 1**.

### Phenotypic Analyses

Embryo sac development and morphology were characterized by cytoembryological observations of cleared ovules following the protocol described by Young et al. (1979). Analysis of *Paspalum* mature embryo sacs at anthesis stage allows the determination of their origin, since meiotic and aposporous embryo sacs display distinct morphologies (Martínez et al., 2001; Ortiz et al., 2013). While mature meiotic embryo sacs (MESs) show an egg cell, one or two synergid cells, one central cell with two polar nuclei and a mass of antipodal cells, aposporous embryo sacs (AESs) are tetra or pentanucleated, and comprise an egg cell, one to two synergids, and two polar nuclei, but lack antipodal cells (Martínez et al., 2001). Spikelets at premeiosis, meiosis or anthesis were fixed in FAA (70% ethanol:formaldehyde:acetic acid 18:1:1) for 24–48 h and transferred to ethanol 70%. Ovaries were then dissected and treated with 3% H_2_O_2_ during 2 h prior to dehydration in an ethanol series (50, 70, 95% and two 100% steps; 30 min each step). Finally, dissected ovaries were cleared using a series of methyl salicylate/ethanol (v/v) solutions (1:1, 3:1, 5.6:1; 30 min each step), incubated in methyl salicylate for at least 12 h, and examined using a Leica DM2500 microscope equipped with Nomarski differential interference contrast (DIC) optics. Seed origin (apomixis vs. sexuality) was determined with the Flow Cytometric Seed Screening (FCSS) method (Matzk et al., 2000), which estimates the genomic DNA content ratio between the embryo and the endosperm (2:3 for sexual seeds; 2:5 for apomictic seeds). Mature seeds were collected 40 days after anthesis. After removing spikelets from the rachis, empty seeds were sorted out using a seed blower. Filled seeds were manually scarified to dissect out caryopses, that were rinsed in a series of 30% v/v hypochlorite, 70% v/v ethanol and sterile distilled water (5 min each step). Then, nuclei were extracted by chopping caryopses into 0.5 ml extraction buffer (CyStain UV Precise P Nuclei extraction buffer, SYSMEX PARTEC, Görlitz, Germany) and filtering through a 50-µm nylon mesh, followed by the addition of 1.5 ml of Cystain UV Precise P Staining Buffer (SYSMEX PARTEC), which contains 4′,6′-diamidino-2-phenylindole (DAPI). The DNA content (C value) was determined by measuring the fluorescence intensity of DAPI-stained nuclei using a CyFlow Space (SYSMEX PARTEC) flow cytometer, following the protocol described by Galdeano el al. (2016). In seed germination experiments, 3 sets of 30 seeds originated from plants E1.4 and E2.9 were scarified with concentrated H_2_SO_4_ during 15 min, rinsed in distilled water and incubated within Petri dishes on wet cotton covered by filter paper, in a growth chamber set at 28°C and a 12h photoperiod for 30 days. Pollen viability was estimated by examining mature pollen grains stained with Alexander’s reagent (Alexander, 1980) using a Nikon Eclipse E200 microscope. Purple-stained grains were scored as viable, while pale-blue/colorless grains indicated sterility.

### Molecular Marker-Based ACR Detection

To verify at the molecular level that the plants transformed with pAct1-F1as did not carry the genomic region responsible for apomixis (ACR), we took advantage of previous works reporting ACR specificity for a 605-bp DNA fragment amplified from a *P. notatum* homolog of a *PEPTIDYL-PROLYL CIS-TRANS ISOMERASE* (*PPIASE*) rice gene (LOC_Os02g52290.1) (Pupilli et al., 2004; Podio et al., 2012). We designed primers to amplify this fragment (**Supplementary Table 1**) and performed PCR experiments in 25 µl reaction mix containing: 90 ng of genomic DNA, 0.2 µM of each primer, 1.5 mM MgCl_2_, 0.2 mM dNTPs and 1.25 U Taq polymerase (INBIO HIGHWAY). Amplification cycles were done in a MyCycler thermal cycler (BIORAD, Hercules, CA, USA), as follows: 5 min at 94°C, 35 cycles of 94°C for 30 s, 59°C for 1 min and 72°C for 2 min, and a final elongation step of 5 min at 72°C. The apomictic accession Q4117 and three apomictic individuals from the JS population (JS-9, JS-71, JS-130), and the sexual accession Q4188 and three sexual individuals from the JS population (JS-36, JS-58, JS-83) were used as positive and negative controls, respectively.

## Results

### Generation of *PN_TGS1*-Like Antisense Lines

**Figure 1:**
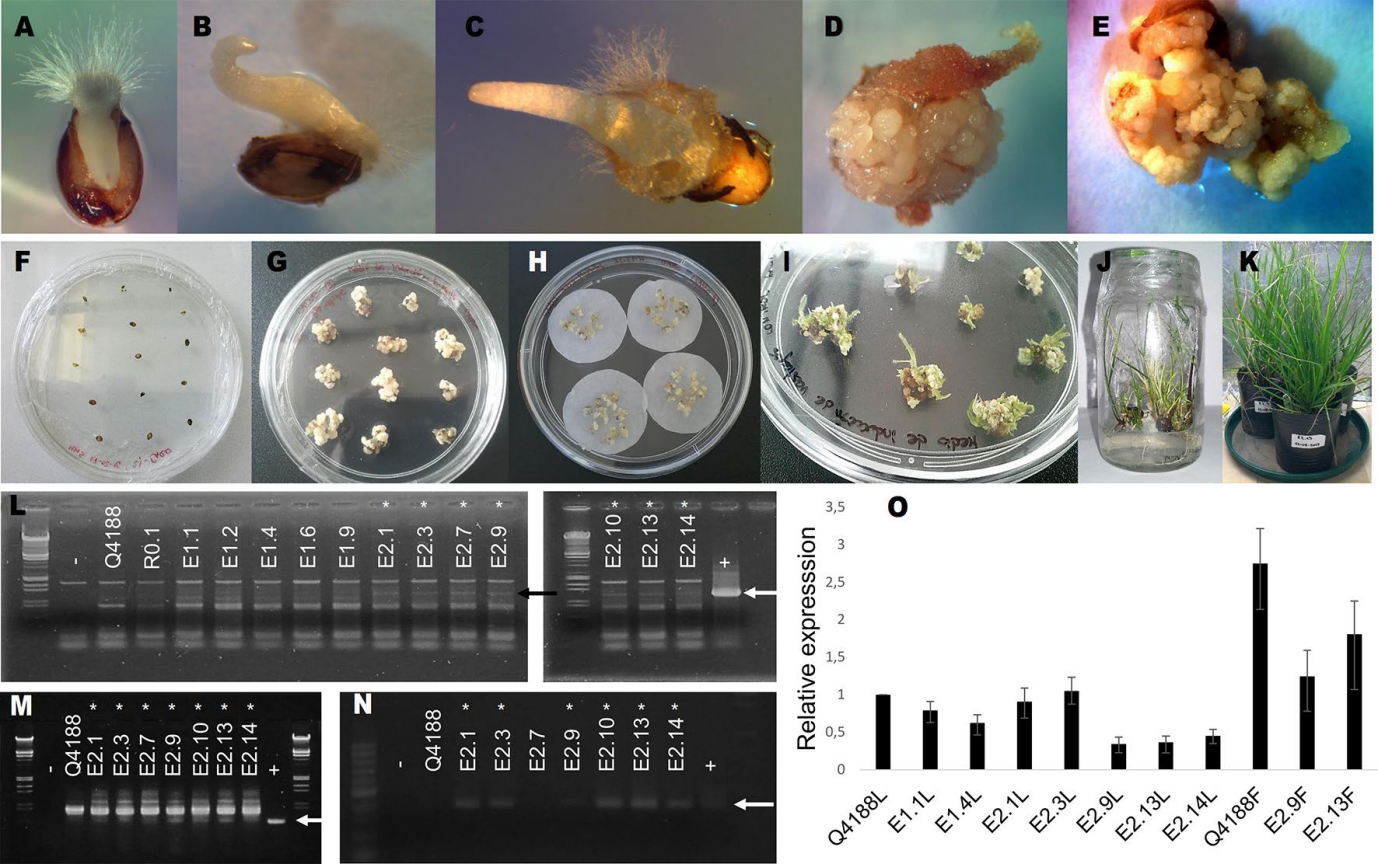
Generation of *PN_TGS1*-like antisense lines by biolistic transformation. **(A**–**E)**: calli induction sequence; mature seeds in MM5 medium. **(A)**: after 3 days. **(B)**: after 8 days. **(C)**: after 10 days. **(D)**:after 21 days (showing calli growth) **(E)**: after 30 days (showing calli growth). **(F**–**K)**: transformation sequence. **(F)**: mature seeds in MM5 induction medium. **(G)**: embryogenic calli before osmotic treatments. **(H)**: embryogenic calli in osmotic medium before bombardment. **(I)**: resistant calli in MRV1 shoot regeneration medium. **(J)**: resistant calli in MEEV3 shoot and root regeneration medium. **(K)**: acclimatized plants. **(L**–**N)**: identification of antisense lines; arrows indicate the expected amplification products. −: negative control. +: positive control. **(L)**: (both left and right panels): amplification with Act-Nos2 Upper/Lower primers. **(M)**: amplification with Act-Nos2 Upper/Nested Lower primers. **(N)**: amplification with NOS Upper/Lower primers. Asterisks indicate positive antisense transgenic lines (E.2.1, E.2.3, E2.7, E.2.9, E.2.10, E.2.13, and E.2.14) **(O)** qRT-PCR experiments for relative quantification of *PN_TGS1*-like expression in leaves **(L)** or flowers **(F)**. Amplification products were generated by using qPCR-Upper/-Lower primers. Q4188: sexual wild type plant. E1.1 and E1.4: transformation controls. E2.1, E2.3, E2.9, E2.13, and E2.14: transgenic (positive) plants.

Co-transformation experiments using pAct1-F1as together with the reporter/selector plasmid pGFPBAR (Huber et al., 2002) allowed the recovery of 12 herbicide-resistant regenerated plants (**Figure 1**). These plants were tested for the presence of the transformation antisense construct using three specific primer pairs (Act-Nos2 Upper/Lower, Act-Nos2 Upper/Nested Lower and NOS Upper/Lower) (**Supplementary Table 1**). Seven plants (E2.1, E2.3, E2.7, E2.9, E2.10, E2.13, and E2.14) amplified the expected bands for primers pairs Act-Nos2 Upper/Lower and Act-Nos2 Upper/Nested Lower. However, only 6 plants showed also amplification with NOS specific primers (E2.1, E2.3, E2.9, E2.10, E2.13, and E2.14), and were therefore selected as true positives. The remaining experimental plants (E1.1, E1.2, E1.4, E1.6, E1.9, and E2.7) were re-classified as transformation controls, since they had gone through the whole transformation procedure, but did not incorporated the pAct1-F1as transformation vector. Then, qPCR experiments were conducted to evaluate *PN_TGS1*-like expression levels in leaves of five transgenic plants out of the total six we had identified (E2.10 did not survive the growth chamber acclimatization period), the E1.1 and E1.4 transformation controls, and Q4188 as wild type sexual control. Three of the transgenic plants (E2.9, E2.13, and E2.14) showed significantly diminished *PN_TGS1*-like expression in leaves compared to wild type and transformation controls (**Figure 1**, **Supplementary Data Sheet 1**), while no significant reduction was detected for E2.1 and E2.3. We further confirmed downregulation of *PN_TGS1*-like in spikelets of E2.9 and E2.13 T_0_ lines (**Figure 1**, **Supplementary Data Sheet 1**) (plant E2.14 has not flowered after 3 years, see below).

### *PN_TGS1* Down-Regulation Induces the Formation of Supernumerary Embryo Sacs

**Figure 2:**
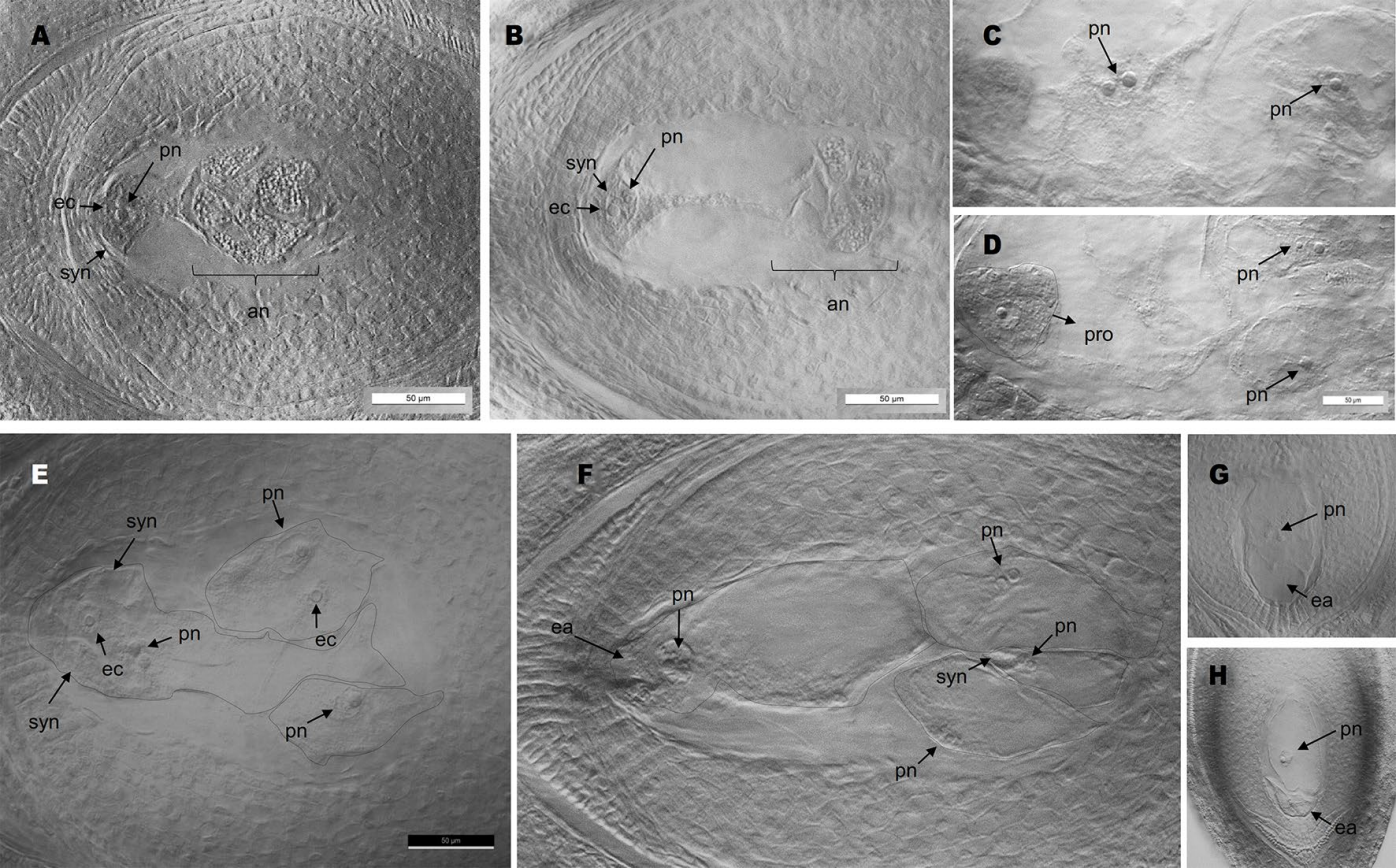
Cytoembryological analysis of antisense and control plants. **(A**, **B)**: meiotic embryo sacs with antipodal cells at the chalazal pole (A: wild type; B: plant E2.9). **(C**, **D)**: adjacent planes of an ovule of obligate aposporous plant Q4117 carrying non-reduced AES; note the coexistence of proembryos arising from parthenogenesis with non-fertilized polar nuclei. **(E**, **F)**: several gametophytes without antipodal cells (AES-like) coexisting in the same ovule (E: plant E2.9; F: plant E2.13) (images of different focal planes are available in **Supplementary Data Sheet 3**). **(G**, **H)**: single AES-like gametophyte without antipodal cells (G: plant E2.13; H: plant E2.9). pn, polar nuclei; syn, synergid cells; ec, egg cell; an, antipodal cells; ea, egg apparatus; pro, proembryo.

**Figure 3:**
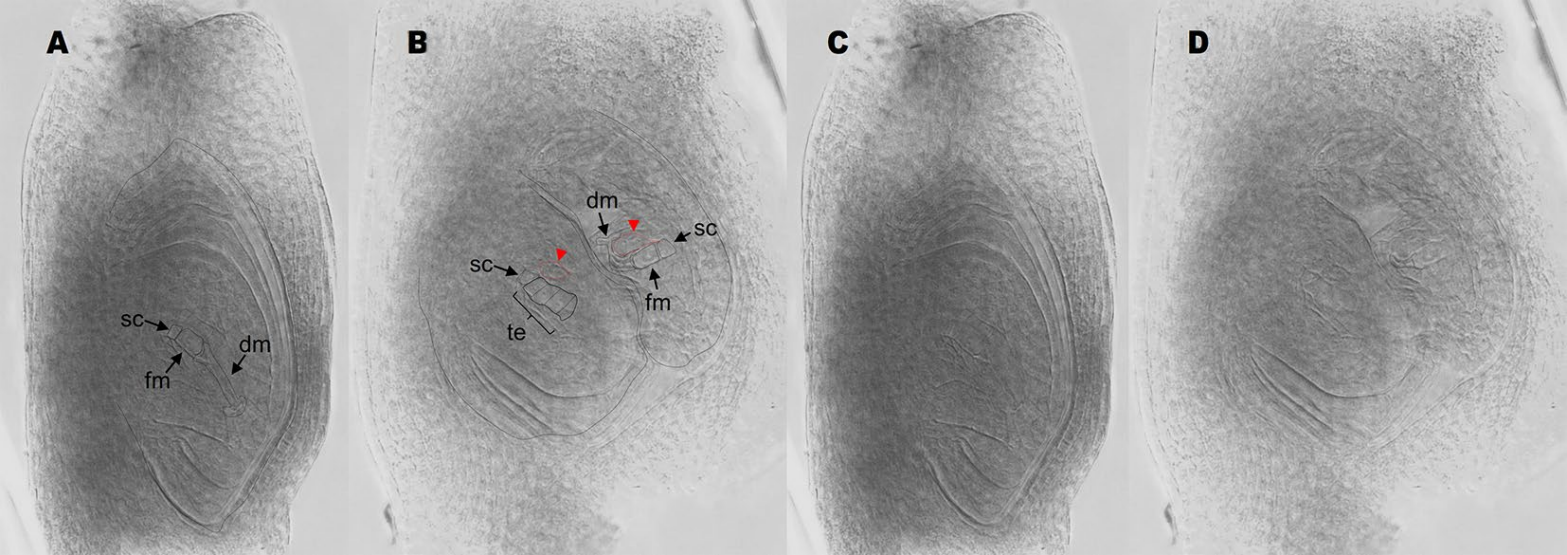
Antisense lines form twin ovules. **(A)** single ovule with a stack cell, a functional megaspore, and degenerating megaspores. **(B)** twin ovules originated from antisense plant E2.9. The ovule on the left bears a stack cell, a meiotic tetrad and two adjacent enlarged nuclei (red arrows/red dotted lines). The ovule on the right shows a stack cell, a functional megaspore, degenerating megaspores and two adjacent enlarged nuclei (red arrows/red dotted lines). **(C**, **D)** same images as in **(A** and **B)**, respectively, but the ovule boundaries were unmarked for an easier viewing of the cell boundaries. sc, stack cell; fm, functional megaspore; dm, degenerated megaspores; te, meiotic tetrad.

Antisense lines (T_0_ generation) showed similar morphological characteristics as controls regarding plant size, vigor and leaf width (see measures of leaf width in **Supplementary Table 2**). However, they displayed long trichomes in the adaxial surface of leaves, a trait that was absent from control plants (**Supplementary Figure 2**, **Supplementary Data Sheet 2**). Moreover, transgenic plants showed delayed flowering: only antisense plants E2.9 and E2.13 flowered in the GMO chamber (see *Materials and Methods*) after 1- and 2-year growing periods, respectively. Antisense plant E2.14 has not flowered yet, after 3 years of vegetative growth. Contrarily, control plants flowered within the first year after being transplanted into soil. A comparative analysis of the number and morphology of inflorescences for antisense lines and control plants is shown in **Supplementary Data Sheet 2**. Plant E2.9 showed a higher number of inflorescences with more than 2 racemes (**Supplementary Data Sheet 2**). Both E2.9 and E2.13 showed shorter racemes with fewer spikelets than control plants (**Supplementary Data Sheet 2**). We found similar proportions of viable and non-viable pollen in *PN_TGS1*-like attenuated plants E2.9 and E2.13, the E1.4 transformation control plant, and the Q4188 wild type plant (**Supplementary Data Sheet 2**). Pollen size was even and similar for antisense and control plants, which suggested absence of non-reduced pollen. On the other hand, cytoembryological analysis at anthesis revealed striking differences between antisense lines and the E1.4 control plant (**Table 1**). All viable ovules of the E1.4 control plant contained a single meiotic embryo sac displaying the characteristic refringent antipodal cells, a pattern highly reminiscent of sexual plant Q4188 reproductive behaviour first reported by Quarin et al. (2003) (**Figure 2**; **Supplementary Figure 3**). Contrarily, a considerable proportion of E2.9 and E2.13 ovules (22.4 and 24.2%, respectively) carried megagametophytes resembling aposporous embryo sacs (AES-like sacs) (33/147 and 23/95 ovules, respectively) (**Figure 2**; **Supplementary Figure 3**; **Supplementary Data Sheet 3**; **Table 1**). In genotypes E.2.9 and E2.13, 34.7% and 42.4% of the ovules carrying AES-like sacs displayed also a MES, respectively, which was usually located in a central position, near the micropyle (**Table 1**). When the AES-like embryo sacs shared the ovule with a legitimate MES embryo sac, they were located to the chalaza, far from the micropyle, as observed for AES of natural apomictic plants. Finally, we observed sporadic occurrence (2.3%) of twin ovules, with one or both bearing MES or AES embryo sacs (**Figure 3**; **Supplementary Figure 4**). No proembryos were detected in either E2.9 or E2.13 ovules at pre-anthesis. In aposporous *Paspalum* species, proembryos are frequently detected at this particular stage, since the egg cells included in the non-reduced embryo sacs are capable to carry on parthenogenesis (**Figure 2**; **Supplementary Figure 3**). The absence of proembryos in E2.9 and E2.13 lines suggests that the AES-like embryo sacs detected in these lines are unable to form embryos by parthenogenesis.

**Table 1:** Embryo sac development assessed by cytoembryological analysis in antisense and control lines at anthesis.

Plant	Number of ovaries	% aborted ovules (n)	% ovules with MES (n)	% ovules with MES + AES-like sacs (n)	% ovules with AES-like sacs only (n)	Proportion of MES/MES + AES-like sacs (95% CI)^a^	Proportion of AES-like/MES + AES-like sacs (95% CI)^b^
E2.9	147	23.13 (34)	54.42 (80)	9.52 (14)	12.93 (19)	0.64 (0.56–0.72)	0.22 (0.16–0.30)
E2.13	95	15.79 (15)	60.00 (57)	8.42 (8)	15.79 (15)	0.68 (0.59–0.77)	0.24 (0.16–0.34)
E1.4	166	12.05 (20)	87.95 (146)	0 (0)	0 (0)	0.88 (0.82–0.92)	0.00 (0–0.03)

a Proportion of ovules carrying MES only + ovules carrying MES and AES-like sacs, followed by the 95% confidence interval including continuity correction (Newcombe, 1998).

b Proportion of ovules carrying AES-like sacs only + ovules carrying MES and AES-like sacs, followed by the 95% confidence interval including continuity correction (Newcombe, 1998). n, number.

**Figure 4:**
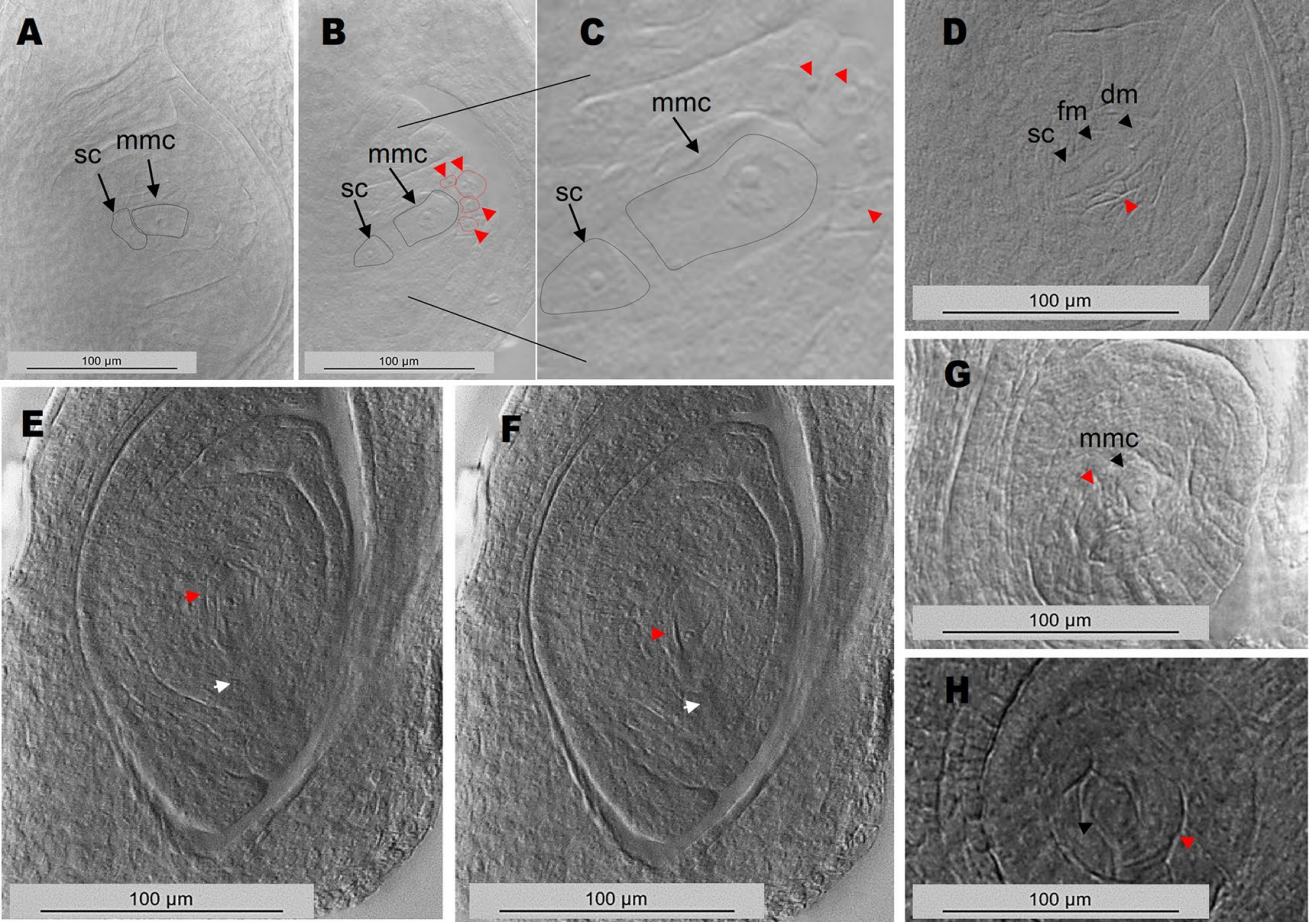
Ovule early developmental stages reveal MMCs and MMC-derived meiotic figures surrounded by cells with enlarged nuclei. **(A)** wild type sexual ovule with a MMC cell and a typical basal stack cell. **(B)** E2.9 antisense transgenic plant ovule showing the MMC, the stack cell and several enlarged nuclei on top (red arrows/red dotted lines). **(C)** same image as in **(B)**, with higher magnification. **(D)** E2.9 antisense transgenic plant ovule with a stack cell, a functional megaspore, degenerating megaspores and an adjacent cell with enlarged nucleus (red arrow). **(E**, **F)** adjacent planes of an ovule containing a two-nucleate gametophyte (fg2) or alternatively a functional megaspore accompanied by a cell with an enlarged nucleus (red arrows). Degenerating megaspores, which should be present at this developmental stage, are absent (the site where degenerating megaspores should have appeared was marked with a white arrow). **(G)** ovule with a megaspore mother cell and a neighbouring cell with enlarged nucleus. **(H)** ovule with a two-nucleate gametophyte (fg2) and a neighbouring cell with enlarged nucleus. mmc, megaspore mother cell; sc, stack cell; fm, functional megaspore; dm, degenerating megaspore.

In order to clarify the origin of the supernumerary embryo sacs detected at anthesis, 80 immature ovules of plants E2.9, Q4188 and E1.4 were examined at early developmental stages (I and II, according to the reproductive calendar of *P. notatum* of Laspina et al., 2008). In the antisense plant E2.9, we detected the presence of MMCs or MMC-derived meiosis products surrounded by nucellar cells with a prominent nucleus and displaying occasionally a knife-shaped morphology. These cells differed from the typical nucellar cells observed in the sexual controls and looked similar to AIs reported for *Paspalum* species (Soliman et al., 2018) (**Figure 4**, **Supplementary Figures 5** and **6**). Twenty-six out of 80 ovules (32.5%, 95% CI: 0.227 < P > 0.44) of E2.9 showed one to several cells with enlarged nuclei surrounding the MMC or the meiotic dyad. None of these AI-like cells seem to initiate meiosis, supporting the hypothesis that the supernumerary embryo sacs observed at anthesis derived from non-reduced cells, as suggested by their morphology. In contrast, ovules of Q4188 and E1.4 sexual plants showed no AIs-like cells (n = 80; 0%, 95% CI: 0 < P > 0.0571), since nucellar cells displayed the typical elongation and nuclei sizes usually detected in sexual plants. Two-nucleate gametophytes (fg2) displaying an aposporous-like morphology were also detected (**Supplementary Figure 6**). Abnormal cells with elongated nuclei were frequently found, but internal vacuoles as well as gametophytes (fg2) were more difficult to identify. Our interpretation is that both immature AES and supernumerary gametophytes of antisense plants occur in all possible orientations, i.e. when early stages ovules are viewed from a sagittal (lateral) plane, MES are detected in frontal morphological planes but AES can be visualized alternatively in sagittal, frontal or transverse perspectives (**Supplementary Figure 6**).

Altogether, our results indicate that *PN_TGS1*-like downregulation in sexual *Paspalum* plants had little effect on somatic development (but for the induction of trichomes in leaves) and male reproduction. In contrast, we observed delayed flowering, anomalous inflorescences, sporadic occurrence of twin ovules, and differentiation of anomalous gametophytes from nucellar cells within ca. 20% of ovules. Among ovules containing atypical gametophytes, a considerable proportion (near 40%) lacked MES, suggesting developmental problems or a difference in competitiveness between both sac types. The absence of proembryos points to a lack of parthenogenesis, since they are commonly detected in aposporous *P. notatum* plants before fertilization of the polar nuclei (pseudogamy).

### Molecular Assay for the Presence of the Apomixis Controlling Region (ACR)

**Figure 5:**
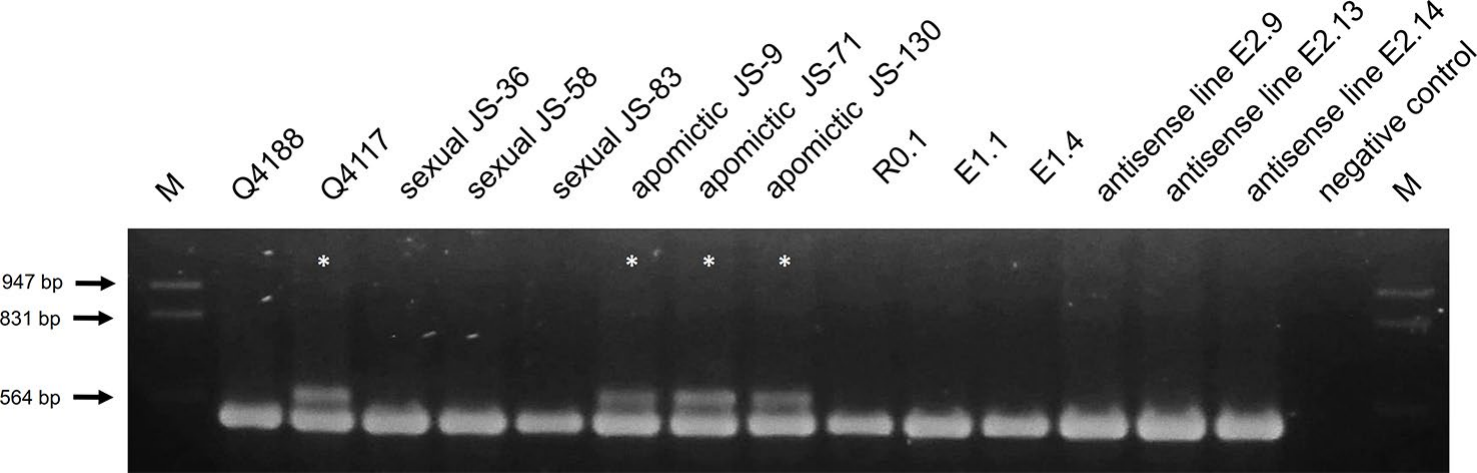
Apospory-controlling region (ACR) amplification in antisense and control lines (sexual and apomictic). Genomic DNA of genotypes Q4188 (sexual), Q4117 (apomictic), three sexual and three apomictic F_1_ hybrids originated from a cross Q4188 x Q4117, R0.1 (regeneration control), E1.1 and E1.4 (transformation controls), and E2.9, E2.13, E2.14 (antisense lines) were used as templates to investigate the presence of the ACR with primers that specifically amplify a copy of a *PPIASE* located within this genomic region (Pupilli et al., 2004; Podio et al., 2012). Only Q4117 and the three apomictic F_1_ hybrids amplified the ACR-specific band.

In order to investigate the presence of the ACR in experimental plants and discard the occurrence of contamination within the sexual seed lot, we checked the antisense lines for a genetic polymorphism of the *PEPTIDYL-PROLYL CIS-TRANS ISOMERASE (PPIASE)* gene, which strictly cosegregates with apomixis in the species (Pupilli et al., 2004; Podio et al., 2012). Using specific primers targeting an internal genomic region of the *P. notatum PPIASE* (**Supplementary Table 1**), we assayed DNA from Q4188 (sexual), Q4117 (apomictic), three apomictic, and three sexual F_1_ hybrids originated from the cross Q4188 x Q4117, the regeneration control R0.1, two transformation controls (E1.1 and E1.4) and three selected antisense lines (E2.9, E2.13 and E2.14). The expected apomixis-linked amplification marker (570 bp) was detected only in Q4117 and the apomictic F1 hybrids (**Figure 5**) and was absent from sexual Q4188, sexual F_1_ progenies and antisense lines (**Figure 5**). This result confirmed that our transgenic lines, derived from sexual seeds, lack the genetic sequence(s) needed for apomixis. Thus, the reproductive phenotypes observed here are expected to derive from down-regulation of the *PN_TGS1*-like gene.

### Antisense Lines Do Not Form Mature Seeds by Apomixis

In order to reconstruct the reproductive developmental pathway of both antisense and control plants, we used the Flow Cytometric Seed Screening (FCSS) method (Matzk et al., 2000) for analysing T_0_ and control seeds. Mature inflorescences were harvested from antisense line E2.9 and E1.4 (transformation control), and empty seeds were sorted out using a wind separator. Both control and antisense lines showed a low seed set (**Table 2**). Such scant values may stem from self-pollination, which reduces seed set in sexual tetraploid germplasm (Zilli et al., 2018), unfavorable greenhouse conditions, the *PN_TGS1* reduced expression, or a combination of all these factors. Examination of nuclear DNA content ratios between the embryo and the endosperm (100 seeds per genotype) allowed to assess whether the seed was generated through sexuality (2C:3C embryo:endosperm ratio), apomixis (2C:5C embryo:endosperm ratio) or fertilization of an unreduced egg cell to produce a BIII individual (3C:5C embryo:endosperm ratio). As expected, the control line (E1.4) formed seeds by sexuality only (**Supplementary Figure 7**). Likewise, all seeds obtained from antisense line E2.9 showed a sexual origin (**Supplementary Figure 7**) and no BIII progeny was detected. These results might support different hypotheses regarding the extra embryo sacs detected at anthesis: a) they are non-functional non-reduced AES-like gametophytes incapable of parthenogenesis or fertilization; b) they are reduced embryo sacs originated from several concurrent meiosis events occurring in the ovule nucellus (less probable, since such simultaneous meiotic events were not detected at early developmental stages); c) they are reduced embryo sacs originated from more than one functional megaspore, derived from a single meiotic event. Out of 30 total seeds (3 replicas, see *Materials and Methods*), an average of 24.66 (± 0.57) and 17.00 (± 1) ones, originated from E1.4 and E2.9, respectively, germinated within the first week. No other seed germinated during the second week. Our results indicate that an inhibition of *PN_TGS1*-like causes a moderate but significant decrease of 25.55% in the percentage of germination of seeds originated by sexuality, since it was 82.22% (72.43 < P> 89.19) for plant E1.4 and 56.67% (45.82 < P> 66.94) for plant E2.9. These results suggest that *PN_TGS1*-like may be involved in embryo/endosperm development and/or the germination pathways during sexual development.

**Table 2:** Proportion of filled caryopses in antisense and control lines.

Plant	Full seeds	Empty seeds	Proportion (95% CI)
Q4188	26	223	0.10 (0.07–0.15)
R0.1	77	733	0.10 (0.07–0.11)
E1.4	51	335	0.13 (0.10–0.17)
E2.9	227	2251	0.09 (0.08–0.10)

## Discussion

PIMT (PRIP-interacting protein with methyltransferase domain)/TGS1 was first isolated as a transcriptional co-activator PRIP-interacting protein (Zhu et al., 2001) and has been extensively studied in yeast, flies and mammals. The canonical PIMT/TGS1 protein contains a methyltransferase and two binding domains, allowing interactions with target RNAs and the methyl donor S-adenosyl-Lmethionine, respectively (Zhu et al., 2001). It participates in several molecular roles affecting growth and development, as follows: 1) catalysing the post-transcriptional conversion of sn(o) and telomerase RNAs 7-methylguanosine caps (m7G) into 2,2,7-trimethylguanosine (m3G), with direct consequences in mRNA splicing, rRNAs processing, and maintenance of the telomere structure (Mouaikel et al., 2002; Franke et al., 2008); 2) modulating transcription in several contexts, by interacting and co-localizing in the nucleus with histone acetyl transferase (HAT)-containing transcriptional coactivators such as CBP/Ep300 and non-HAT-containing coactivators such as the Mediator subunit Med1 (PPAR binding protein; PBP/TRAP220/DRIP205) and PRIP (Zhu et al., 2001; Misra et al., 2002; Kornberg, 2007). PIMT has been proposed to serve as a molecular bridge between HAT- and non-HAT-containing transcriptional complexes and to control nuclear receptor mediated transcription. ERK2 phosphorylation at Ser^298^ of PIMT/TGS1 activates transcriptional activity at some promoters, suggesting a direct role for signal transduction pathways in modulating transcription (Kapadia et al., 2013); 3) trimethylating the 7-methylguanosine caps (m7G) of a group of quiescence-induced primary miRNAs (pri-miRNAs), which bind Exportin-1 in primary human fibroblasts (Martínez et al., 2017). Knockdown of either Exportin-1 or TGS1 inhibits the biogenesis of the corresponding mature miRNAs (Martínez et al., 2017). These Exportin-1–dependent pri-miRNAs are found in the cytoplasm during quiescence together with a smaller isoform of Drosha (Martínez et al., 2017). It was proposed that in quiescent cells an alternative miRNA biogenesis pathway involving TGS1 and Exportin-1 selectively processes and transports a specific set of miRNAs, which could be essential for reversible G0 arrest (Martínez et al., 2017).

In contrast, TGS1 remains poorly characterized in plants. Interestingly, these organisms evolved a longer gene family member, including an N-terminal extension with a WW protein-interacting domain (Siena et al., 2014), which suggests the rise of plant-specific functions. In sub-tropical *P. notatum* plants, the plant-specific gene version *PN_TGS1*-like expresses throughout the female reproductive development in nucellar and integument cells of ovules in sexual biotypes, while no expression is detected in ovules of apomictic plants (Siena et al., 2014). Moreover, *PN_TGS1*-like expression positively correlates with the rate of sexuality in facultative biotypes (Siena et al., 2014). Interestingly, *PN_TGS1*-like is naturally downregulated only in ovules, since no significant differential expression was detected in leaves or roots between apomictic and sexual genotypes (Siena et al., 2014). Recently it was shown that *TGS1*-like is required for plant chilling tolerance, since the vegetative and reproductive growth of *Arabidopsis tgs1-like* mutants (with defective AT1G45231 expression) was severely compromised under this particular condition (Gao et al., 2017).

In order to obtain antisense lines with a reduced *PN_TGS1*-like expression, we first isolated a specific gene fragment (named F1) located at the 5′ end of the transcript, and absent from other partially homologous sequences. F1 was cloned under the rice Act1 promoter control, which allows constitutive expression in all Gramineae plant organs, including ovules (Zhang et al., 1991; Mancini et al., 2014; Mancini et al., 2018). Three antisense lines with reduced *PN_TGS1*-like grew normally at 26°C, showing leaf development, color, and shape indistinguishable from that of the wild type. However, long trichomes formed in the adaxial surface of leaves and were visually distinguishable. The emergence of the latter trait (which is absent from both sexual and apomictic wild type plants) might be associated with the repression of *PN_TGS1*-like in the leaves of the transgenic plants due to the use of the constitutive rice Act1 promoter. In apomictic genotypes, *PN_TGS1*-like is downregulated only in ovules, while expression in leaves is equivalent to that observed in sexual plants (Siena et al., 2014). This might account for the absence of long trichomes in the leaf adaxial surface of both sexual and apomictic plants. Besides, downregulation of *PN_TGS1*-like caused the occasional emergence of abnormal gametophytic nuclei representation in mature ovules. These atypical nuclei might be associated with to the occurrence of one to several gametophytes with an aposporous-like morphology and/or represent a misguided differentiation process, involving proliferation and/or changes of the cellular developmental fate. Unfortunately, reliable methods aimed at estimating the ploidy level within the female gametophyte are not available for *Paspalum*. However, analyses of reproductive female development at early premeiotic/meiotic developmental stages revealed the occurrence of enlarged cells with prominent nuclei adjacent to the MMC or the meiotic products, which were never seen to initiate meiosis. The absence of simultaneous meiotic events in the antisense plants immature ovules supports a non-reduced nature for the extra nuclei detected at anthesis stage, but the development of more than one functional megaspore from the MMC meiosis cannot be discarded. None of the transformed (antisense) lines carried the sequence/s necessary for apomixis in its genome, as was demonstrated by the absence of an ACR-specific *PPIASE* marker. Therefore, the emergence of the reproductive phenotype can be assigned to the downregulation of *PN_TGS1*-like gene only, and not to an inherent capacity for apomictic reproduction. No proembryos were observed at preanthesis/anthesis stage, and seed flow cytometry analyses revealed that only the sexual reproductive pathway was active in transgenic plants. The two last findings point to the absence of parthenogenesis and/or endosperm development. However, further experiments will be needed to test this hypothesis, like control of proembryo formation after emasculation and crossing of antisense *PN_TGS1*-like with endosperm autonomous development mutant/transformant lines, among others.

If the extra embryo sacs detected in the transgenic plants E2.9 and E2.13 were unreduced (as suggested by their morphology), and their egg cells were incapable of parthenogenesis (as suggested by the sexual origin of the whole E2.9 progeny), an increased formation of BIII (2n + n) progeny should be expected (Martínez et al., 1994; Espinoza et al., 2002). However, the proportion of BIII hybrids formation in *Paspalum* is influenced by the pollination time (Martínez et al., 1994; Espinoza et al., 2002). Pollination at anthesis (the stage used here to produce the seed set) generates an insignificant proportion of BIII progeny (1/97, Proportion 0.0103, 95% CI: 0.0005 < P< 0.0642, see Espinoza et al., 2002), even in full apomicts, in which the percentage of ovules carrying unreduced embryo sacs is close to 100%. The rate of BIII formation in complete absence of parthenogenesis was never analysed, since in *P. notatum* apospory and parthenogenesis were never uncoupled. The only record reporting an increase of the BIII formation rate associate to an induced decrease of parthenogenesis was presented by Podio et al. (2014b), who used 5-azacytidine treatments to de-methylate the whole *Paspalum* genome. Podio et al. (2014b) reported that from 33 non-maternal progenies, 8 were BIII hybrids (24.24%, 95% CI: 0.1174 < P < 0.4263); here we analysed 100 ovaries, from which none was a BIII hybrid (0%, 95% CI: 0.000 < P < 0.0461). Data sets are significantly different, but consideration should be paid to the fact that the natural apomictic plants used by Podio et al. (2014b) were obligate apomictic genotypes forming unreduced female gametes in near all ovules, while the transgenic antisense plants studied here have only 22.45–24.21% of ovules carrying aposporous embryo sacs. Further flow cytometry analyses involving a higher number of ovules should be planned. Moreover, we cannot discard that a modification of *TGS1* expression could affect fertilization of unreduced gametes.

Out of the few apomixis candidate genes previously identified by comparative transcriptomics/genomics/reverse genetics, only the MAP3K QUI-GON JINN (QGJ), seems to be involved in the development of aposporous embryo sacs (Mancini et al., 2018). Both *PN_TGS1*-like and PN*_QGJ* are expressed in the *P. notatum* ovule nucellus at premeiotic stage (Siena et al., 2014; Mancini et al., 2018), but have opposite expression level depending on the genotype reproductive mode: *PN_TGS1*-like is overexpressed in sexual genotypes with respect to aposporous ones, while *PN_QGJ* is upregulated in aposporous genotypes with respect to sexual ones. Interestingly, downregulation of *PN_QGJ* impairs the formation of AES in the nucellus of an obligate apomictic genotype (Mancini et al., 2018). On the contrary, here we show that downregulation of *PN_TGS1*-like induces the formation of AES-like in the nucellus of sexual genotypes. These results suggest the existence of two antagonistic pathways related with the megagamethogenesis operating in the *Paspalum* ovule nucellus, one of them promoting apospory (expressed only in aposporous plants) and the other one repressing it (expressed only in sexual plants). The connection between these pathways could be further analysed by determining the expression of *PN_QGJ* in antisense *PN_TGS1*-like lines and vice versa. Crosses of the antisense/RNAi lines could be used to analyse which of these controlling molecules is dominant. Since both RNAi *PN_QGJ* and antisense *PN_TGS1*-like plants are available in our laboratory, we are able to carry out these crosses, in order to initiate the study of functional links operating between the sexual and aposporous molecular pathways.

There is only one previous report (Zhao et al., 2017) that identifies a molecular pathway controlling the number of mature embryo sacs formed within the plant ovule. Zhao et al. (2017) determined that CDK-inhibitors of the KRP class are expressed within MMCs in order to restrict the CDKA1-dependent inactivation of the *Arabidopsis* RETINOBLASTOMA homolog RBR1. One function of RBR1 is the direct repression of the stem cell factor WUSCHEL (WUS), a process necessary to allow the entry of the MMC into a meiotic fate. In *rbr*1 and *krp* triple mutants, WUS ectopically accumulates in the designated meiocytes, inducing them to undergo several mitotic divisions before initiating meiosis. Consequently, several meioses occur in parallel, and multiple reduced embryo sacs are formed within the ovule. Depleting WUS in *rbr1* mutants restores the formation of only a single meiocyte, and a single derived embryo sac. Interestingly, the egg/central cell of the supernumerary reduced embryo sacs formed in *rbr*1 and *krp* triple mutants partially fail to undergo plasmogamy with sperm cells, suggesting that the KRP-controlled molecular pathway is also involved in the fertilization process (Zhao et al., 2017). Like RBR1 and KRP, *PN_TGS1*-like seems to control the number of embryo sacs formed in the ovule, but the mechanism involved is dissimilar, since in this instance AIs-like cells emerge from the nucellus and multiple meioses are not detected. Therefore, RBR1/KRP and *PN_TGS1*-like seem to trigger different controlling mechanisms, equally necessary to ensure that the sexually reproducing plant will carry only a single embryo next to the nourishing tissue (i.e., the endosperm). Meanwhile, a regulatory mechanism similar to that reported here for *PN_TGS1*-like has been proposed in ARGONAUTE 9 (AGO9) defective mutants (Olmedo-Monfil et al., 2010). However, *ago9* mutants do not form complete mature supernumerary embryo sacs, even when several germline cells differentiate from nucellar cells. In the near future, it will be of interest to explore the functional link between *AGO9* and *PN_TGS1*-like, which could be related with the already reported *TGS1* activity during miRNA trimethylation (Martínez et al., 2017).

Apomixis has for long been considered a valuable trait from a plant breeding perspective, since it could allow the fixation of hybrid vigor (Hanna, 1995). In fact, the combined use of apomixis and sexuality in breeding programs allows rapid generation of superior genotypes, whose heterosis can be permanently fixed and inherited *via* seeds (Spillane et al., 2004). Currently, such type of breeding schemes are being applied to improve natural apomictic forage grasses of the *Paspalum* and *Brachiaria* genera in South and North America (Acuña et al., 2007; Acuña et al., 2009; Quesenberry et al., 2010; Acuña et al., 2011; Barrios et al., 2013). The success of this methodology has accelerated the production of adapted hybrid cultivars and is rapidly boosting livestock farming in areas that had traditionally been marginal for cattle production. Moreover, advanced molecular strategies have been developed to allow the production of artificial clonal seeds, mimicking the consequences of apomictic development (Khanday et al., 2019; Wang et al., 2019). The concept that apomixis could be induced in sexual major crops species gets even more attractive when considering recently posed hypotheses pointing to both reproductive modes being anciently polyphenic (Albertini et al., 2019), since identification of a lost developmental switch could allow changing from one reproductive route to the other at will. In this context, the identification and characterization of molecules like *PN_TGS1*-like, which can be used alone or in combination to induce particular alterations in gametophyte development, represents an important step towards the effective modulations of the reproductive mode with consequences for breeding.

## Data Availability Statement

Most datasets generated for this study are included in the article/**Supplementary Material**. Any other raw data supporting the conclusions of this manuscript will be made available by the authors, without undue reservation, to any qualified researcher.

## Author Contributions

CC collaborated in transformation experiments, identified the transformed plants, and carried out expression analyses and phenotypic characterization. JO participated of the experimental design, and collaborated in antisense phenotypic analysis and in manuscript writing. HP and ES performed transformation experiments. LS cloned the *PN_TGS1*-like antisense fragment, collaborated in phenotypic characterization and contributed to the establishment of the plant material. NS carried out the PPIase validation experiments. FG and FE conducted the flow cytometry analyses. OL participated in the experimental design and the writing of the manuscript. SP designed the experiments, supervised and participated in the experimental work, and wrote the article. All authors agree to be accountable for the content of the work.

## Funding

The European Union’s Horizon 2020 Research and Innovation Programme under the Marie Skłodowska-Curie Grant Agreement No 645674; Agencia Nacional de Promoción Científica y Tecnológica (ANPCyT), Argentina, Projects PICT-2014-1080 and PICT-2017-1956; Consejo Nacional de Investigaciones Científicas y Técnicas (CONICET), Argentina, Projects: PIP 11220090100613 and PUE 22920160100043CO; Universidad Nacional de Rosario (UNR), Argentina, Project: AGR271. CC and NS received fellowships from CONICET. LS, JO, FE and SP are research staff members of CONICET.

## Conflict of Interest

The authors declare that the research was conducted in the absence of any commercial or financial relationships that could be construed as a potential conflict of interest.

## References

[B1] Acuña C. A. Blount A.R. Quesenberry K.H. Hanna W.W. Kenworthy K.E. (2007). Reproductive characterization of bahiagrass germplasm. Crop Sci. 47, 1711-1717.

[B2] Acuña C. A. Blount A. R. Quesenberry K. H. Kenworthy K. E. Hanna W. W. (2009). Bahiagrass tetraploid germplasm: reproductive and agronomic characterization of segregating progeny. Crop Sci. 49, 581–588. 10.2135/cropsci2008.07.0402

[B3] Acuña C. A. Blount A. R. Quesenberry K. H. Kenworthy K. E. Hanna W. W. (2011). Tetraploid bahiagrass hybrids: breeding technique, genetic variability and proportion of heterotic hybrids. Euphytica 179, 227–235. 10.1007/s10681-010-0276-y

[B4] Akiyama Y. Conner J. A. Goel S. Morishige D. T. Mullet J. E. Hanna W. W. (2004). High-resolution physical mapping in *Pennisetum squamulatum* reveals extensive chromosomal heteromorphism of the genomic region associated with apomixis. Plant Physiol. 134, 1733–1741. 10.1104/pp.103.033969 15064383 PMC419846

[B5] Albertini E. Marconi G. Barcaccia G. Raggi L. Falcinelli M. (2004). Isolation of candidate genes for apomixis in *Poa pratensis* . Plant Mol. Biol. 56, 879–894. 10.1007/s11103-004-5211-y 15821987

[B6] Albertini E. Barcaccia G. Carman J. G. Pupilli F. (2019). Did apomixis evolve from sex or was it the other way around? J. Exp. Bot. 70, 2951–2964. 10.1093/jxb/erz109 30854543

[B7] Alexander M. P. (1980). Differential staining of aborted and non-aborted pollen. Stain Technol. 44, 117–122. 10.3109/10520296909063335 4181665

[B8] Barrios S. C. L. Do Valle C. B. Alves G. F. Simeão R. M. Jank L. (2013). Reciprocal recurrent selection in the breeding of *Brachiaria decumbens* . Trop. Grasslands-Forrajes Trop. 1, 52–54. 10.17138/TGFT(1)52-54

[B9] Calderini O. Chang S. B. de Jong H. Busti A. Paolocci F. Arcioni S. (2006). Molecular cytogenetics and DNA sequence analysis of an apomixis-linked BAC in *Paspalum simplex* reveal a non pericentromere location and partial microcolinearity with rice. Theor. Appl. Genet. 112, 1179–1191. 10.1007/s00122-006-0220-7 16463157

[B10] Calderini O. Donnison I. Polegri L. Panara F. Thomas A. Arcioni S. (2011). Partial isolation of the genomic region linked with apomixis in *Paspalum simplex* . Mol. Breed. 28, 265–276. 10.1007/s11032-010-9480-7

[B11] Conner J. A. Goel S. Gunawan G. Cordonnier-Pratt M. M. Johnson V. E. Liang C. (2008). Sequence analysis of bacterial artificial chromosome clones from the apospory-specific genomic region of *Pennisetum* and *Cenchrus* . Plant Physiol. 147, 1396–1411. 10.1104/pp.108.119081 18508959 PMC2442526

[B12] Conner J. A. Mookkan M. Huo H. Chae K. Ozias-Akins P. (2015). A parthenogenesis gene of apomict origin elicits embryo formation from unfertilized eggs in a sexual plant. P. Natl. Acad. Sci. U.S.A. 112, 11205–11210. 10.1073/pnas.1505856112 PMC4568661 26305939

[B13] Crane C. F. (2001). “Classification of apomictic mechanisms,” in The flowering of apomixis: from mechanisms to genetic engineering. Eds. Savidan Y. Carman J. G. Dresselhaus T. (Mexico City: CIMMYT, IRD, European Commission DG VI FAIR), 24–43.

[B14] Espinoza F. Pessino S. C. Quarin C. L. Valle E. M. (2002). Effect of pollination timing on the rate of apomictic reproduction revealed by RAPD markers in *Paspalum notatum* . Ann. Bot. 89, 165–170. 10.1093/aob/mcf024 12099347 PMC4233789

[B15] Felitti S. A. Seijo J. G. González A. M. Podio M. Laspina N. V. Siena L. (2011). Expression of *LORELEI*-like genes in aposporous and sexual *Paspalum notatum* plants. Plant Mol. Biol. 77, 337–354. 10.1007/s11103-011-9814-9 21826430

[B16] Franke J. Gehlen J. Ehrenhofer-Murray A. E. (2008). Hypermethylation of yeast telomerase RNA by the snRNA and snoRNA methyltransferase TGS1. J. Cell. Sci. 121, 3553–3560. 10.1242/jcs.033308 18840651

[B17] Galdeano F. Urbani M. H. Sartor M. E. Honfi A. I. Espinoza F. Quarin C. L. (2016). Relative DNA content in diploid, polyploid, and multiploid species of *Paspalum* (Poaceae) with relation to reproductive mode and taxonomy. J. Plant Res. 129, 697–710. 10.1007/s10265-016-0813-4 26965283

[B18] Gao J. Wallis J. G. Jewell J. B. Browse J. (2017). Trimethylguanosine synthase 1 (TGS1) is essential for chilling tolerance. Plant Physiol. 174, 1713–1727. 10.1104/pp.17.00340 28495891 PMC5490903

[B19] Hand M. Koltunow A. (2014). The genetic control of apomixis: asexual seed formation. Genetics 197, 441–450. 10.1534/genetics.114.163105 24939990 PMC4063905

[B20] Hanna W. W. (1995). Use of apomixis in cultivar development. Adv. Agron. 54, 333–350. 10.1016/S0065-2113(08)60903-7

[B21] Hojsgaard D. Klatt S. Baier R. Carman J. G. Hörandl E. (2014). Taxonomy and biogeography of apomixis in angiosperms and associated biodiversity characteristics. Crit. Rev. Plant Sci. 33, 414–427. 10.1080/07352689.2014.898488 27019547 PMC4786830

[B22] Huber M. Hahn R. Hess D. (2002). High transformation frequencies obtained from a commercial wheat (*Triticum aestivum* L. cv. ‘Combi’) by microbombardment of immature embryos followed by GFP screening combined with PPT selection. Mol. Breed. 10, 19–30. 10.1023/A:1020338632330

[B23] Kapadia B. Viswakarma N. Parsa K. V. L. Kain V. Behera S. Suraj S. K. (2013). ERK2-mediated phosphorylation of transcriptional coactivator binding protein PIMT/ NcoA6IP at Ser298 augments hepatic gluconeogenesis. PloS One 8, e83787. 10.1371/journal.pone.0083787 24358311 PMC3866170

[B24] Khanday I. Skinner D. Yang B. Mercier R. Sundaresan V. (2019). A male-expressed rice embryogenic trigger redirected for asexual propagation through seeds. Nature 565, 91–95. 10.1038/s41586-018-0785-8 30542157

[B25] Kornberg R. D. (2007). The molecular basis of eukaryotic transcription. P. Natl. Acad. Sci. U.S.A. 104, 12955–12961. 10.1073/pnas.0704138104 PMC1941834 17670940

[B26] Laspina N. V. Vega T. Martelotto L. Stein J. Podio M. Ortiz J. P. (2008). Gene expression analysis at the onset of aposporous apomixis in *Paspalum notatum* . Plant Mol. Biol. 67, 615–628. 10.1007/s11103-008-9341-5 18481185

[B27] Leon-Martínez G. Vielle-Calzada J. P. (2019). Apomixis in flowering plants: developmental and evolutionary considerations. Curr. Top. Dev. Biol. 131, 565–604. 10.1016/bs.ctdb.2018.11.014 30612631

[B28] Mancini M. Woitovich N. Permingeat H. Podio M. Siena L. A. Ortiz J. P. A. (2014). Development of a modified transformation platform for apomixis candidate genes research in *Paspalum notatum* (bahiagrass). In Vitro. Cell. Dev. PL. 50, 412–424. 10.1007/s11627-014-9596-2

[B29] Mancini M. Permingeat H. Colono C. Siena L. Pupilli F. Azzaro C. (2018). The MAP3K-coding *QUI-GON JINN* (*QGJ*) gene is essential to the formation of unreduced embryo sacs in *Paspalum* . Front. Plant Sci. 9, 1547. 10.3389/fpls.2018.01547 30405677 PMC6207905

[B30] Martínez E. J. Espinoza F. Quarin C. L. (1994). BIII progeny (2n + n) from apomictic *Paspalum notatum* obtained through early pollination. J. Hered. 85, 295–297. 10.1093/oxfordjournals.jhered.a111460

[B31] Martínez E. J. Urbani M. H. Quarin C. L. Ortiz J. P. A. (2001). Inheritance of apospory in bahiagrass, *Paspalum notatum* . Hereditas 135, 19–25. 10.1111/j.1601-5223.2001.00019.x 12035611

[B32] Martínez I. Hayes K. E. Barr J. A. Harold A. D. Xie M. Bukhari S. I. A. (2017). An Exportin-1–dependent microRNA biogenesis pathway during human cell quiescence. Proc. Natl. Acad. Sci. U. S. A. 114, E4961–E4970. 10.1073/pnas.1618732114 28584122 PMC5488920

[B33] Matzk F. Meister A. Schubert I. (2000). An efficient screen for reproductive pathways using mature seeds of monocots and dicots. Plant J. 21, 97–108. 10.1046/j.1365-313x.2000.00647.x 10652155

[B34] Misra P. Qi C. Yu S. Shah S. H. Cao W. Q. Sambasiva Rao M. (2002). Interaction of PIMT with transcriptional coactivators CBP, p300, and PBP differential role in transcriptional regulation. J. Biol. Chem. 277, 20011–20019. 10.1074/jbc.M201739200 11912212

[B35] Mouaikel J. Verheggen C. Bertrand E. Tazi J. Bordonné R. (2002). Hypermethylation of the cap structure of both yeast snRNAs and snoRNAs requires a conserved methyltransferase that is localized to the nucleolus. Mol. Cell. 9, 891–901. 10.1016/S1097-2765(02)00484-7 11983179

[B36] Newcombe R. G. (1998). Two-sided confidence intervals for the single proportion: comparison of seven methods. Stat. Med. 17, 857–872. 10.1002/(SICI)1097-0258(19980430)17:8<857::AID-SIM777>3.0.CO;2-E 9595616

[B37] Nogler G. A. (1984). “Gametophytic Apomixis,” in Embryology of Angiosperms. Ed. Johri B. M. (Berlin, Germany: Springer-Verlag), 475–518. 10.1007/978-3-642-69302-1_10

[B38] Ochiai-Fukuda T. Takahashi-Ando N. Ohsato S. Igawa T. Kadokura K. Hamamoto H. (2006). A fluorescent antibiotic resistance marker for rapid production of transgenic rice plants. J. Biotechnol. 122, 521–527. 10.1016/j.jbiotec.2005.09.015 16271791

[B39] Ochogavía A. C. Seijo J. G. González A. M. Podio M. Laspina N. V. Duarte Silveira E. (2011). Characterization of retrotransposon sequences expressed in inflorescences of apomictic and sexual *Paspalum notatum* plants. Sex Plant Reprod. 24, 231–246. 10.1007/s00497-011-0165-0 21394488

[B40] Okada T. Hu Y. Tucker M. R. Taylor J. M. Johnson S. D. Spriggs A. (2013). Enlarging cells initiating apomixis in *Hieracium praealtum* transition to an embryo sac program prior to entering mitosis. Plant Physiol. 163, 216–231. 10.1104/pp.113.219485 23864557 PMC3762643

[B41] Olmedo-Monfil V. Durán-Figueroa N. Arteaga-Vázquez M. Demesa-Arévalo E. Autran D. Grimanelli D. (2010). Control of female gamete formation by a small RNA pathway in *Arabidopsis* . Nature 464, 628–632. 10.1038/nature08828 20208518 PMC4613780

[B42] Ortiz J. P. A. Pessino S. C. Leblanc O. Hayward M. D. Quarin C. L. (1997). Genetic fingerprint for determinig the mode of reproduction in *Paspalum notatum*, a subtropical apomictic forage grass. Theor. Appl. Genet. 95, 850–856. 10.1007/s001220050635

[B43] Ortiz J. P. A. Quarin C. L. Pessino S. C. Acuña C. Martínez E. J. Espinoza F. (2013). Harnessing apomictic reproduction in grasses: what we have learned from *Paspalum* . Ann. Bot. 112, 767–787. 10.1093/aob/mct152 23864004 PMC3747805

[B44] Ortiz J. P. A. Revale S. Siena L. A. Podio M. Delgado L. Stein J. (2017). A reference floral transcriptome of sexual and apomictic *Paspalum notatum* . BMC Genomics 18, 318. 10.1186/s12864-017-3700-z 28431521 PMC5399859

[B45] Ortiz J. P. A. Leblanc O. Rohr C. Grisolia M. Siena L. A. Podio M. (2019). Small RNA-seq reveals novel regulatory components for apomixis in *Paspalum notatum* . BMC Genomics 20, 487. 10.1186/s12864-019-5881-0 31195966 PMC6567921

[B46] Paterson A. H. Brubaker C. L. Wendel J. F. (1993). A rapid method for extraction of cotton (*Gossypium* spp.) genomic DNA suitable for RFLP or PCR analysis. Plant Mol. Biol. Rep. 11, 122–127. 10.1007/BF02670470

[B47] Pessino S. C. Espinoza F. Martínez E. J. Ortiz J. P. A. Valle E. M. Quarin C. L. (2001). Isolation of cDNA clones differentially expressed in flowers of apomictic and sexual *Paspalum notatum* . Hereditas 134, 35–42. 10.1111/j.1601-5223.2001.00035.x 11525063

[B48] Podio M. Rodríguez M. P. Felitti S. Stein J. Martínez E. Siena L. A. (2012). Sequence characterization, in silico mapping and cytosine methylation analysis of markers linked to apospory in *Paspalum notatum* . Genet. Mol. Biol. 35, 827–837. 10.1590/S1415-47572012005000070 23271945 PMC3526092

[B49] Podio M. Felitti S. A. Siena L. A. Delgado L. Mancini M. Seijo G. (2014a). Characterization and expression analysis of SOMATIC EMBRYOGENESIS RECEPTOR KINASE (SERK) genes in sexual and apomictic *Paspalum notatum* . Plant Mol. Biol. 84, 479–495. 10.1007/s11103-013-0146-9 24146222

[B50] Podio M. Cáceres M. E. Samoluk S. Seijo J. G. Pessino S. C. Ortiz J. P. A. (2014b). A methylation status analysis of the apomixis-specific region in *Paspalum* spp. suggests an epigenetic control on parthenogenesis. J. Exp. Bot. 10.1093/jxb/eru354 25180110

[B51] Polegri L. Calderini O. Arcioni S. Pupilli F. (2010). Specific expression of apomixis-linked alleles revealed by comparative transcriptomic analysis of sexual and apomictic *Paspalum simplex* Morong flowers. J. Exp. Bot. 61, 1869–1883. 10.1093/jxb/erq054 20231327

[B52] Pupilli F. Martínez E. J. Busti A. Calderini O. Quarin C. L. Arcioni S. (2004). Comparative mapping reveals partial conservation of synteny at the apomixis locus in *Paspalum* spp. Mol. Genet. Genomics 270, 539–548. 10.1007/s00438-003-0949-5 14648202

[B53] Qiu Z. R. Shuman S. Schwer B. (2011). An essential role for trimethylguanosine RNA caps in *Saccharomyces cerevisiae* meiosis and their requirement for splicing of SAE3 and PCH2 meiotic pre-mRNAs. Nucleic Acids Res. 39, 5633–5646. 10.1093/nar/gkr083 21398639 PMC3141232

[B54] Quarin C. L. (1999). Effect of pollen source and pollen ploidy on endosperm formation and seed set in pseudogamous apomictic Paspalum notatum. Sex Plant Reprod. 11, 331–335.

[B55] Quarin C. L. Espinoza F. Martínez E. J. Pessino S. C. Bovo O. A. (2001). A rise of ploidy level induces the expression of apomixis in *Paspalum notatum* . Sex Plant Reprod. 13, 243–249. 10.1007/s004970100070

[B56] Quarin C. L. Urbani M. H. Blount A. R. Martínez E. J. Hack C. M. Burton G. W. (2003). Registration of Q4188 and Q4205, sexual tetraploid germplasm lines of bahiagrass. Crop Sci. 43, 745–746. 10.2135/cropsci2003.0745

[B57] Quesenberry K. H. Dampier J. M. Lee Y. Y. Smith R. L. Acuña C. A. (2010). Doubling the chromosome number of bahiagrass via tissue culture. Euphytica. 175, 43–50. 10.1007/s10681-010-0165-4

[B58] Rodrigues J. C. Cabral G. B. Dusi D. M. A. Mello L. V. Rinden D. Carneiro V. T. C. (2003). Identification of differentially expressed cDNA sequences in ovaries of sexual and apomictic plants of *Brachiaria brizantha* . Plant Mol. Biol. 53, 745–757. 10.1023/B:PLAN.0000023664.21910.bd 15082923

[B59] Sambrook J. Russell D. W. (2001). Molecular cloning- A laboratory manual (New York: Cold Spring Harbor Laboratory Press).

[B60] Siena L. A. Ortiz J. P. A. Leblanc O. Pessino S. (2014). *PNTGS1*-like expression during reproductive development supports a role for RNA methyltransferases in the aposporous pathway. BMC Plant Biol. 14, 297. 10.1186/s12870-014-0297-0 25404464 PMC4243328

[B61] Siena L. A. Ortiz J. P. A. Calderini O. Paolocci F. Cáceres M. E. Kaushal P. (2016). An apomixis-linked ORC3-like pseudogene is associated with silencing of its functional homolog in apomictic *Paspalum simplex* . J. Exp. Bot. 67, 1965–1978. 10.1093/jxb/erw018 26842983

[B62] Spillane C. Curtis M. D. Grossniklaus U. (2004). Apomixis technology development-Virgin births in farmers' fields? Nat. Biotechnol. 22, 687–691. 15175691 10.1038/nbt976

[B63] Soliman M. Espinoza F. Ortiz J. P. A. Delgado L. (2018). Heterochronic reproductive developmental processes between diploid and tetraploid cytotypes of *Paspalum rufum* . Ann. Bot. 123, 901–915. 10.1093/aob/mcy228 PMC6526369 30576402

[B64] Stein J. Quarin C. L. Martínez E. J. Pessino S. C. Ortiz J. P. A. (2004). Tetraploid races of *Paspalum notatum* show polysomic inheritance and preferential chromosome pairing around the apospory-controlling locus. Theor. Appl. Genet. 109, 186–191. 10.1007/s00122-004-1614-z 14985979

[B65] Stein J. Pessino S. C. Martínez E. J. Rodríguez M. P. Siena L. A. Quarin C. L. (2007). A genetic map of tetraploid *Paspalum notatum* Flügge (bahiagrass) based on single-dose molecular markers. Mol. Breed. 20, 153–166. 10.1007/s11032-007-9083-0

[B66] Wang C. Qing L. Shen Y. Hua Y. Wand J. Lin J. (2019). Clonal seeds from hybrid rice by simultaneous genome engineering of meiosis and fertilization genes. Nat. Biotechnol. 37, 283–286. 10.1038/s41587-018-0003-0 30610223

[B67] Yamada-Akiyama H. Akiyama Y. Ebina M. Xu Q. Tsuruta S. Yazaki J. (2009). Analysis of expressed sequence tags in apomictic Guinea grass (*Panicum maximum*). J. Plant Physiol. 166, 750–761. 10.1016/j.jplph.2008.10.001 19046615

[B68] Young B. A. Sherwood R. T. Bashaw E. C. (1979). Cleared-pistil and thick-sectioning techniques for detecting aposporous apomixis in grasses. Can. J. Bot. 57, 1668–1672. 10.1139/b79-204

[B69] Zhang W. McElroy D. Wu R. (1991). Analysis of rice actl 50 region activity in transgenic rice plants. Plant Cell 3, 1155–1165. 1821763 10.1105/tpc.3.11.1155 PMC160082

[B70] Zhao X. A. Bramsiepe J. Van Durme M. Komaki S. Prusicki M. A. Maruyama D. (2017). RETINOBLASTOMA RELATED1 mediates germline entry in *Arabidopsis* . Science 356, eaaf6532. 10.1126/science.aaf6532 28450583

[B71] Zhu Y. Qi C. Cao W. Q. Yeldandi A. V. Rao M. S. Reddy J. K. (2001). Cloning and characterization of PIMT, a protein with a methyltransferase domain, which interacts with and enhances nuclear receptor coactivator PRIP function. P. Natl. Acad. Sci. U.S.A. 98, 10380–10385. 10.1073/pnas.181347498 PMC56969 11517327

[B72] Zilli A. L. Brugnoli E. A. Marcón F. Billa M. B. Ríos E. F. Martínez E. J. (2015). Heterosis and expressivity of apospory in tetraploid bahiagrass hybrids. Crop Sci. 55, 1189–1201. 10.2135/cropsci2014.10.0685

[B73] Zilli A. L. Acuña C. A. Schulz R. R. Brugnoli E. A. Guidalevich V. Quarin C. L. (2018). Widening the gene pool of sexual tetraploid bahiagrass: generation and reproductive characterization of a sexual synthetic tetraploid population. Crop Sci. 58, 762–772. 10.2135/cropsci2017.07.0457

