# Supplementary material for: A Plant-Specific TGS1 Homolog Influences Gametophyte Development in Sexual Tetraploid Paspalum notatum Ovules
Source: Front Plant Sci. 2019 Nov 29;10:1566. doi: 10.3389/fpls.2019.01566 (PMC6895069; doi:10.3389/fpls.2019.01566)
Supplement: Supplementary file 1 [file DataSheet_1.pdf]

Supplementary Data Sheet 1

PN_TGS1_LIKE EXPRESSION IN LEAVES

E1.1 vs WT (Q4188)

Relative Expression Results

Parameter	Value
Iterations	2000

Gene	Type	Reaction Effic	Expression	Std. Error	95% C.I.	P(H1)	Result
TGS1	TRG	0,7608	0,791	0,671 - 0,951	0,601 - 1,052	0,01	DOWN
Tubuline	REF	0,8692	1				

Interpretation

TGS1 is DOWN-regulated in sample group (in comparison to control group) by a mean factor of 0,791 (S.E. range is 0,671 - 0,951).

TGS1 sample group is different to control group. P(H1)=0,005

E2.1 vs WT (Q4188)

Relative Expression Results

Parameter	Value
Iterations	2000

Gene	Type	Reaction Effic	Expression	Std. Error	95% C.I.	P(H1)	Result
TGS1	TRG	0,77	0,908	0,729 - 1,129	0,584 - 1,270	0,32	
Tubuline	REF	0,8408	1				

Interpretation

TGS1 sample group is not different to control group. P(H1)=0,320

E2.3 vs WT (Q4188)

Relative Expression Results

Parameter	Value
Iterations	2000

Gene	Type	Reaction Effic	Expression	Std. Error	95% C.I.	P(H1)	Result
TGS1	TRG	0,7467	1,049	0,865 - 1,224	0,800 - 1,441	0,54	
Tubuline	REF	0,84	1				

Interpretation

TGS1 sample group is not different to control group. P(H1)=0,535

E2.9 vs WT (Q4188)

Relative Expression Results

Parameter	Value
Iterations	2000

Gene	Type	Reaction Effic	Expression	Std. Error	95% C.I.	P(H1)	Result

TGS1	TRG	0,7717	0,343	0,252 - 0,459	0,224 - 0,555	0	DOWN
Tubuline	REF	0,8633	1				

Interpretation

TGS1 is DOWN-regulated in sample group (in comparison to control group) by a mean factor of 0,343 (S.E. range is 0,252 - 0,459).

TGS1 sample group is different to control group. P(H1)=0,001

E2.13 vs WT (Q4188)

Relative Expression Results

Parameter	Value
Iterations	2000

Gene	Type	Reaction Effic	Expression	Std. Error	95% C.I.	P(H1)	Result
TGS1	TRG	0,8242	0,365	0,280 - 0,505	0,237 - 0,675	0	DOWN
Tubuline	REF	0,8408	1				

Interpretation

TGS1 is DOWN-regulated in sample group (in comparison to control group) by a mean factor of 0,365 (S.E. range is 0,280 - 0,505).

TGS1 sample group is different to control group. P(H1)=0,000

E2.14 vs WT (Q4188)

Relative Expression Results

Parameter	Value
Iterations	2000

Gene	Type	Reaction Effic	Expression	Std. Error	95% C.I.	P(H1)	Result
TGS1	TRG	0,7975	0,453	0,369 - 0,556	0,328 - 0,630	0	DOWN
Tubuline	REF	0,7967	1				

TGS1 is DOWN-regulated in sample group (in comparison to control group) by a mean factor of 0,453 (S.E. range is 0,369 - 0,556).

TGS1 sample group is different to control group. P(H1)=0,000

Supplementary Data Sheet 1

PN_TGS1_LIKE EXPRESSION IN FLOWERS

E2.9 vs WT (Q4188)

Relative Expression Results

Parameter	Value
Iterations	2000

Gene	Type	Reaction Effic	Expression	Std. Error	95% C.I.	P(H1)	Result
TGS1	TRG	0,7558	0,51	0,362 - 0,678	0,235 - 0,752	0,002	DOWN
Tubuline	REF	0,7333	1				

Interpretation

TGS1 is DOWN-regulated in sample group (in comparison to control group) by a mean factor of 0,510 (S.E. range is 0,362 - 0,678).

TGS1 sample group is different to control group. P(H1)=0,002

E2.13 vs WT (Q4188)

Relative Expression Results

Parameter	Value
Iterations	2000

Gene	Type	Reaction Effic	Expression	Std. Error	95% C.I.	P(H1)	Result
TGS1	TRG	0,8667	0,568	0,425 - 0,824	0,367 - 0,996	0	DOWN
Tubuline	REF	0,7475	1				

Interpretation

TGS1 is DOWN-regulated in sample group (in comparison to control group) by a mean factor of 0,568 (S.E. range is 0,425 - 0,824).

TGS1 sample group is different to control group. P(H1)=0,000
