# Supplementary material for: A Plant-Specific TGS1 Homolog Influences Gametophyte Development in Sexual Tetraploid Paspalum notatum Ovules
Source: Front Plant Sci. 2019 Nov 29;10:1566. doi: 10.3389/fpls.2019.01566 (PMC6895069; doi:10.3389/fpls.2019.01566)
Supplement: Supplementary file 2 [file DataSheet_2.pdf]

Supplementary Data Sheet 2

Number of trichomes formed in transformed and control plants

**Plant**	**Total number of cm examined**	**Total number of trichomes scored**	**Trichomes/cm**	**SD[a]**
WT (Q4188)(control)	14	0	0	0
E1.4 (control)	23	0	0	0
E2.9 (antisense)	66	166	2.51	0.74
E2.13 (antisense)	28	79	2.8	1.19
E2.14 (antisense)	33	181	5.48	1.44

[a] Standard deviation

Inflorescences number and morphology

**Plant**	**Number of inflorescences analyzed**	**Racemes per inflorescence (SD)[a]**	**Number of spikelets per row (SD)[a]**	**Raceme length (cm) (SD)[a]**
WT (Q4188)	12	2 (0)	48.5 (12.0)	13.7 (1.6)
E1.4 (control)	8	2 (0)	42.1 (7.6)	12.23 (2.9)
E2.9 (antisense)	45	2.7 (0.5)	32.6 (7.1)	9.92 (2.0)
E2.13 (antisense)	6	2 (0)	33.5 (1.9)	10.41 (0.5)

[a] Standard deviation

Pollen viability analysis

**Plant**	**Number of VP**	**Number of NVP**	**Total**	**%NVP (95% CI)**
WT	5191	3250	8441	38.5 (37.5<P> 39.6)
E1.4	4047	2080	6127	34.0 (32.8<P> 35.2)
E2.9	3519	2421	5940	40.76 (39.51<P> 42.02)
E2.13	3464	2368	5832	40.60 (39.34<P> 41.87)

VP: viable pollen, NVP: non-viable pollen, % NVP: percentage of non-viable pollen
