# Supplementary material for: A Plant-Specific TGS1 Homolog Influences Gametophyte Development in Sexual Tetraploid Paspalum notatum Ovules
Source: Front Plant Sci. 2019 Nov 29;10:1566. doi: 10.3389/fpls.2019.01566 (PMC6895069; doi:10.3389/fpls.2019.01566)
Supplement: Supplementary file 3 [file DataSheet_3.pdf]

Supplementary Data Sheet 3

Adjacent focal planes of the antisense plants’ ovules shown in Figure 2E-F and ovules of sexual and apomictic controls

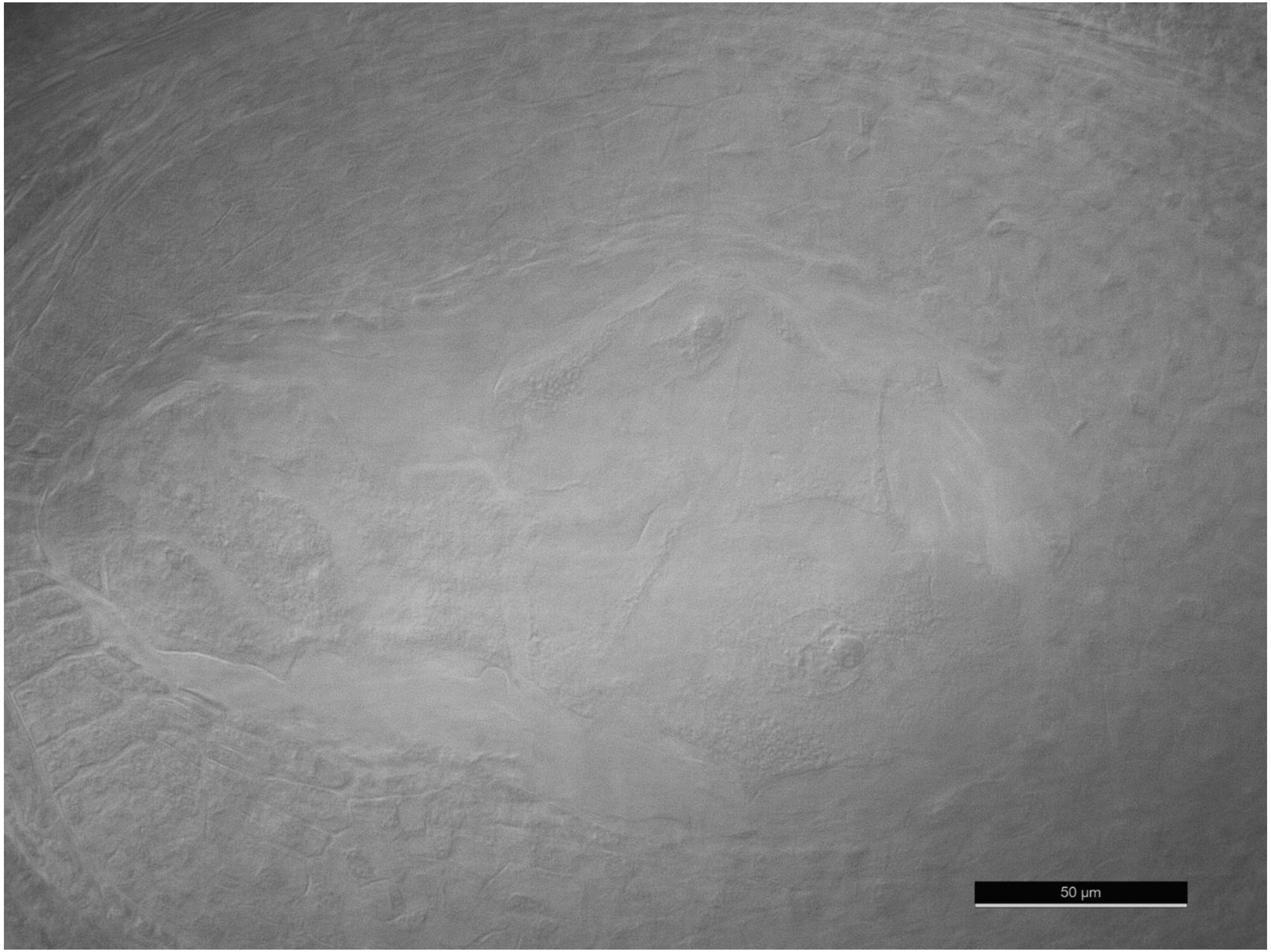

Fig. 2E ovule, antisense plant E2.9, focal plane 1

Fig. 2E ovule, antisense plant E2.9, focal plane 2

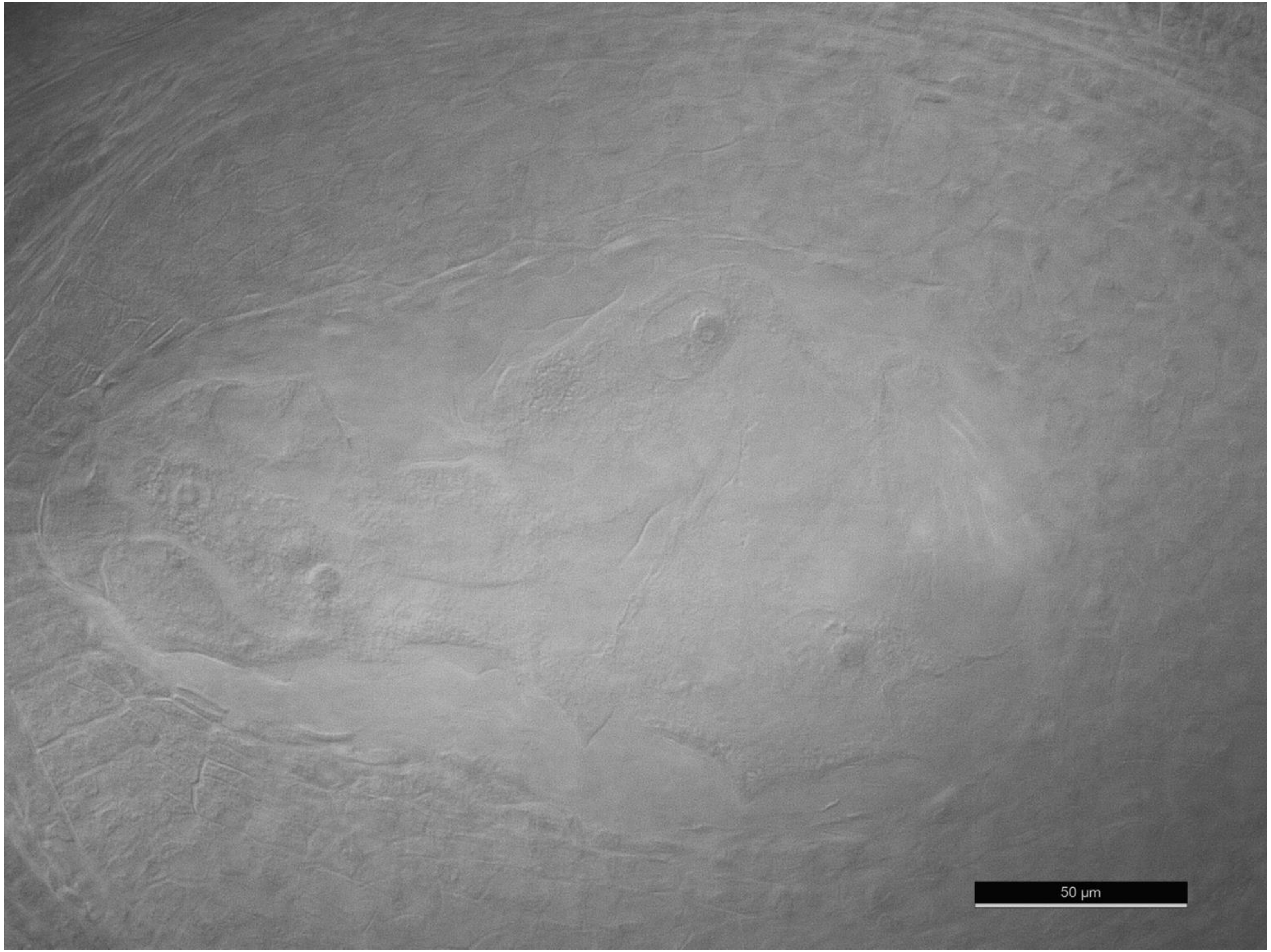

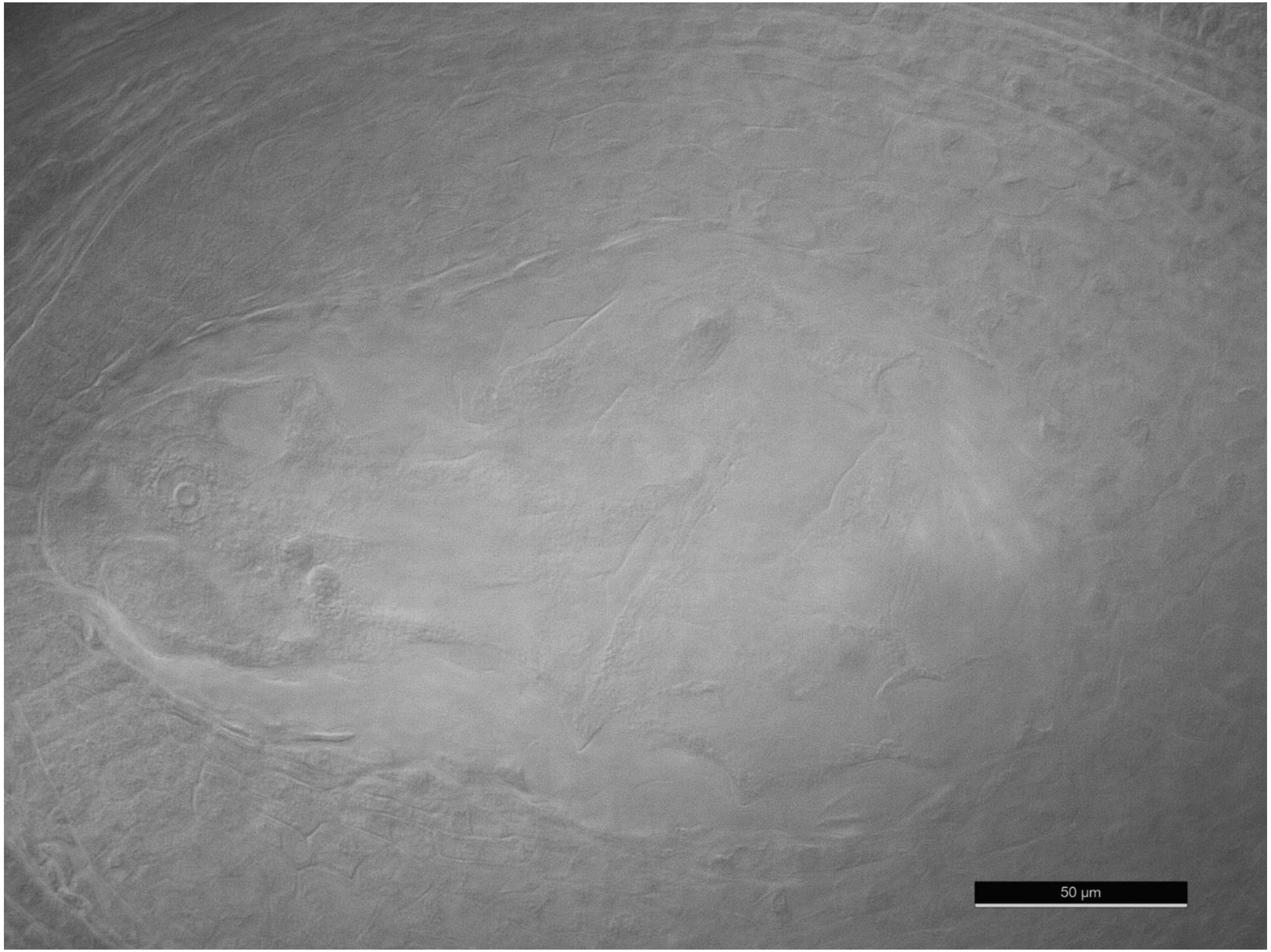

Fig. 2E ovule, antisense plant E2.9, focal plane 3

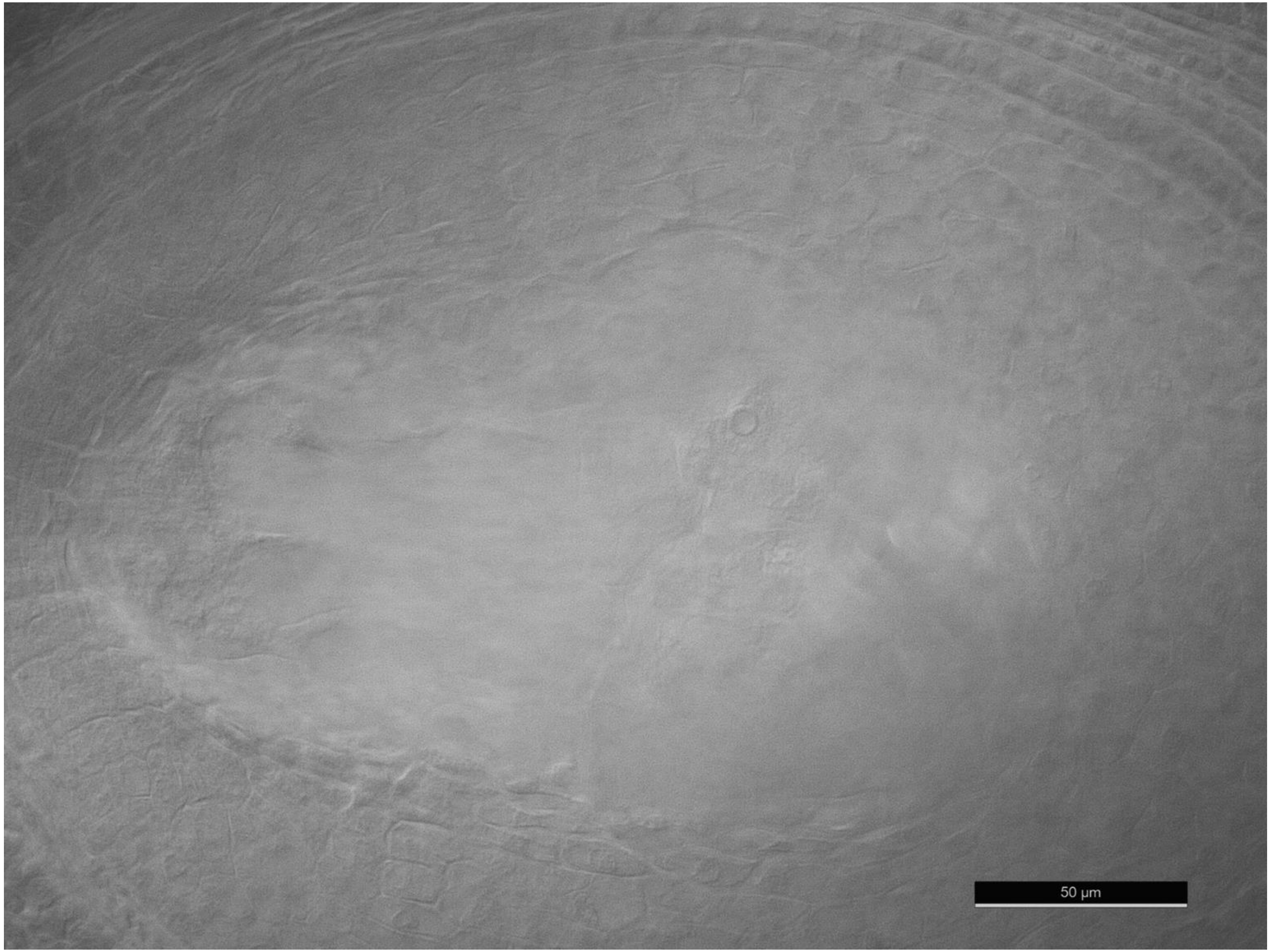

Fig. 2E ovule, antisense plant E2.9, focal plane 4

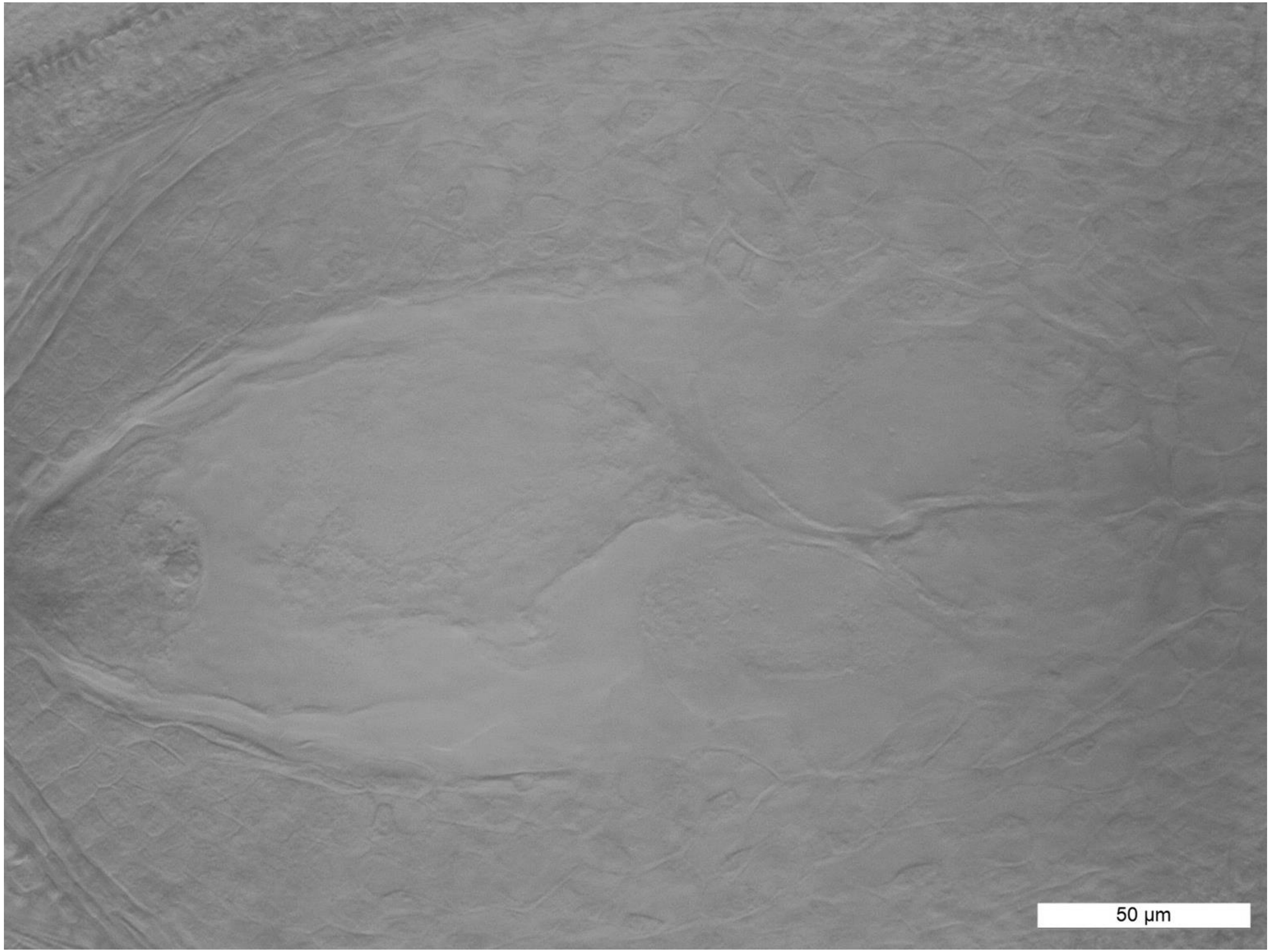

Fig. 2F ovule, antisense plant E2.13, focal plane 1

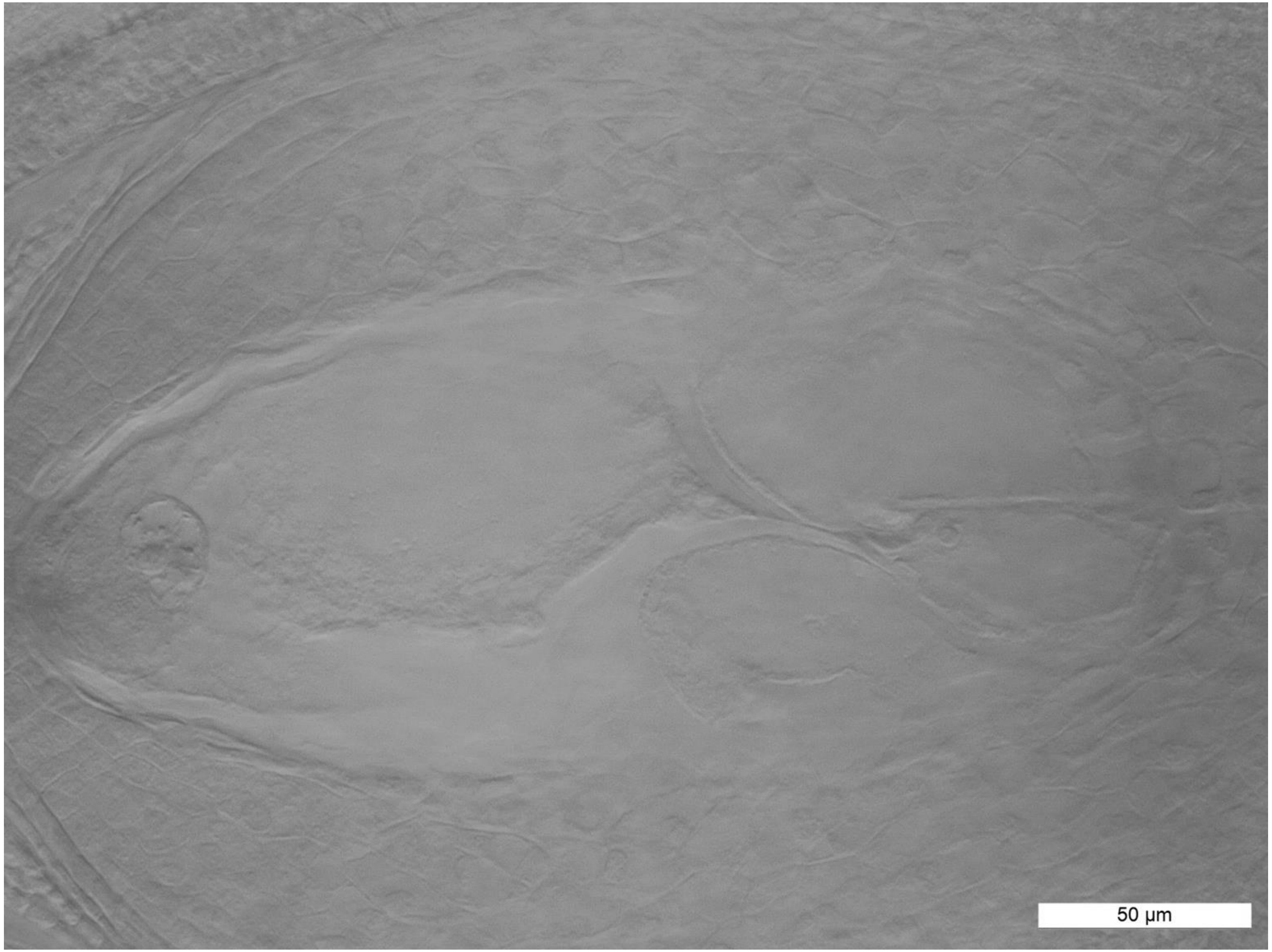

Fig. 2F ovule, antisense plant E2.13, focal plane 2

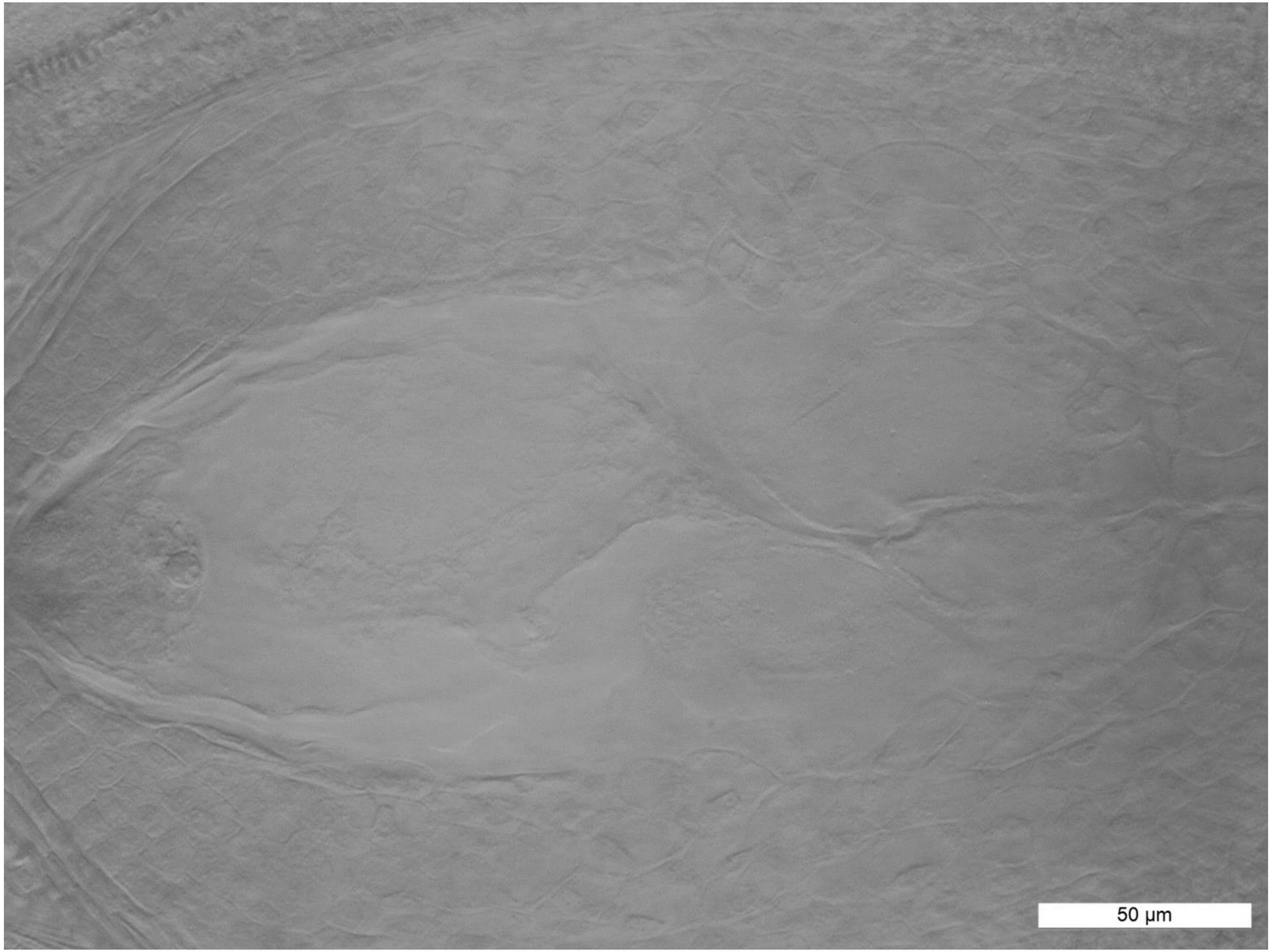

Fig. 2F ovule, antisense plant E2.13, focal plane 3

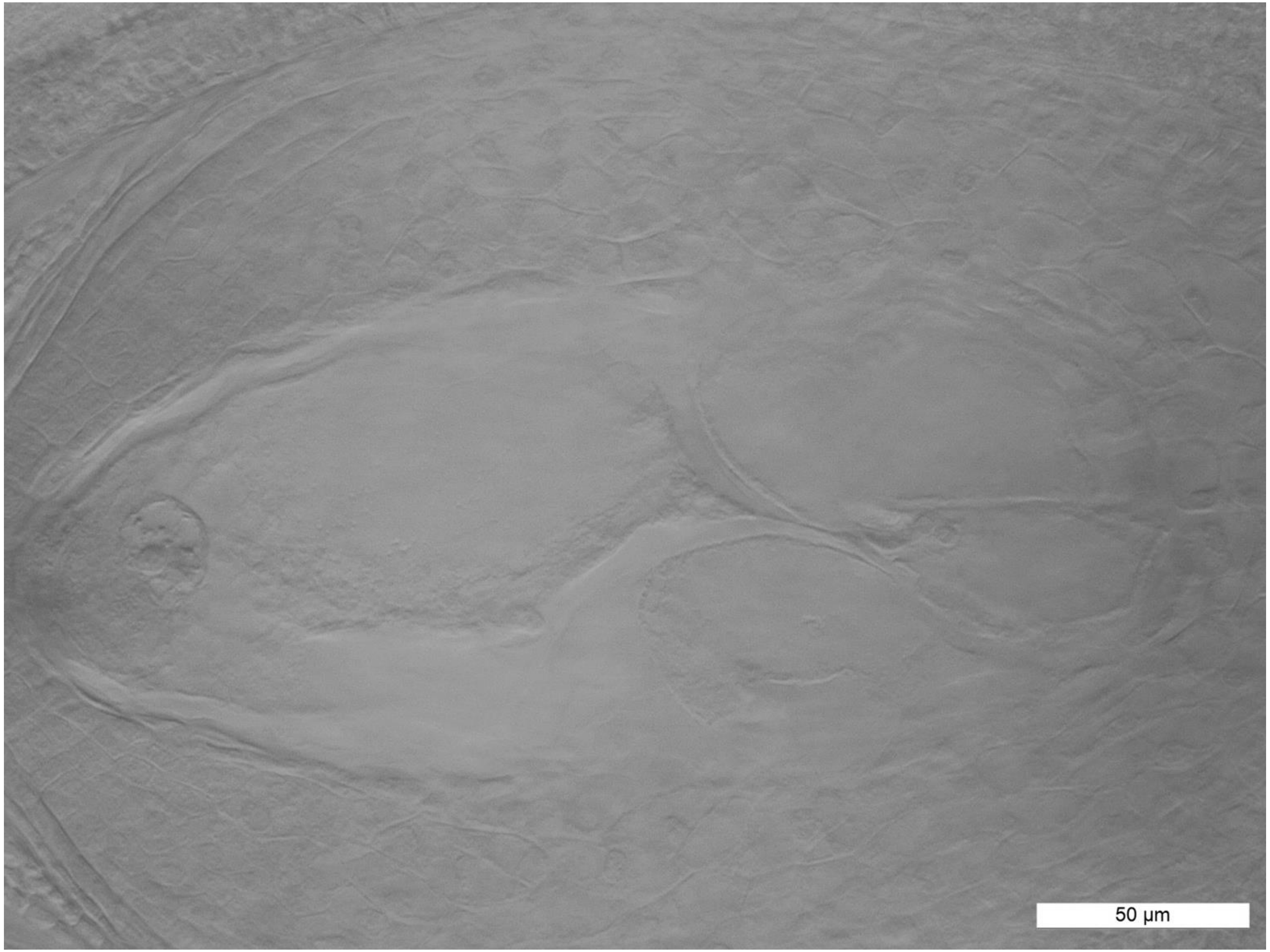

Fig. 2F ovule, antisense plant E2.13, focal plane 4

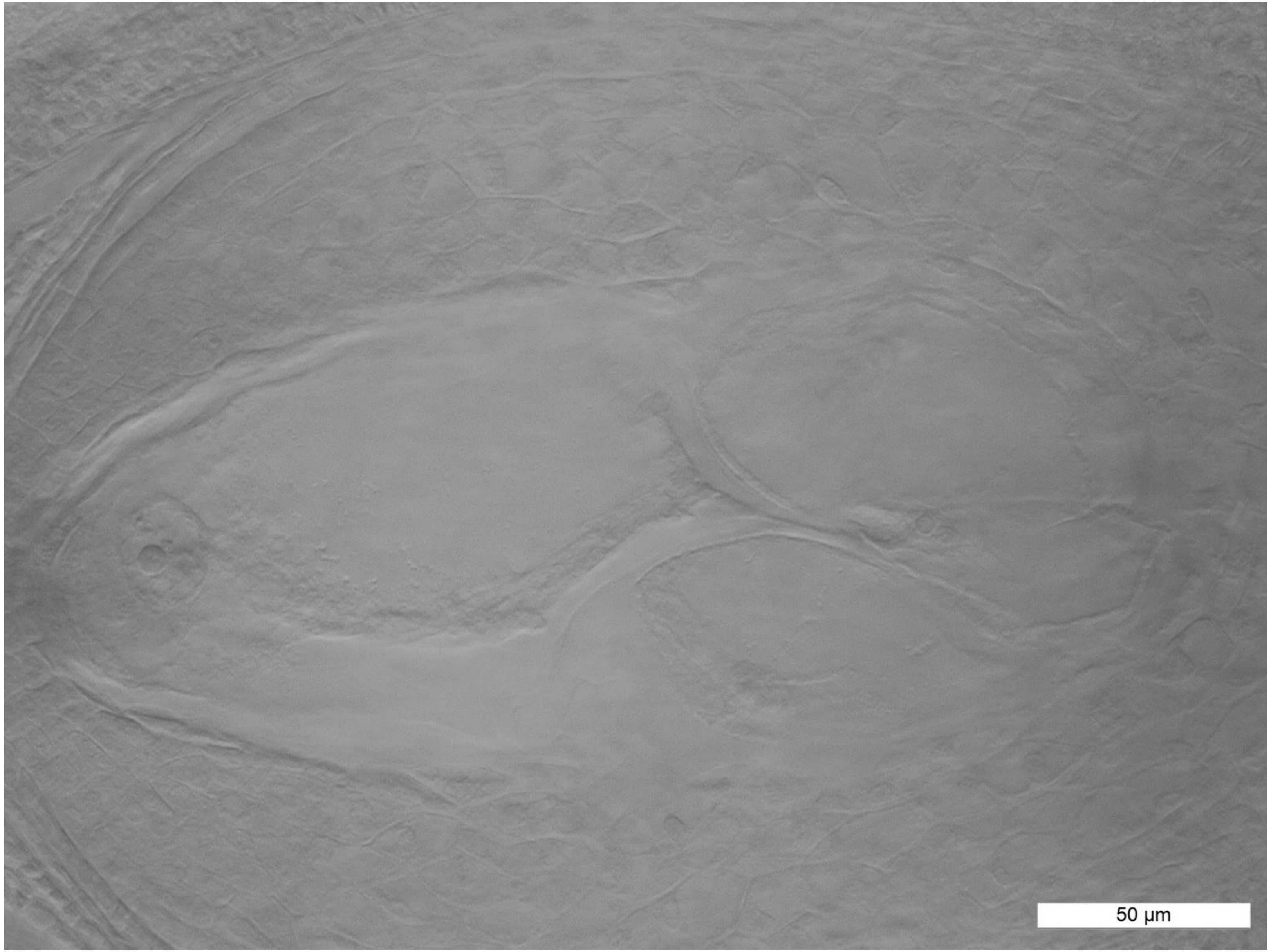

Fig. 2F ovule, antisense plant E2.13, focal plane 5

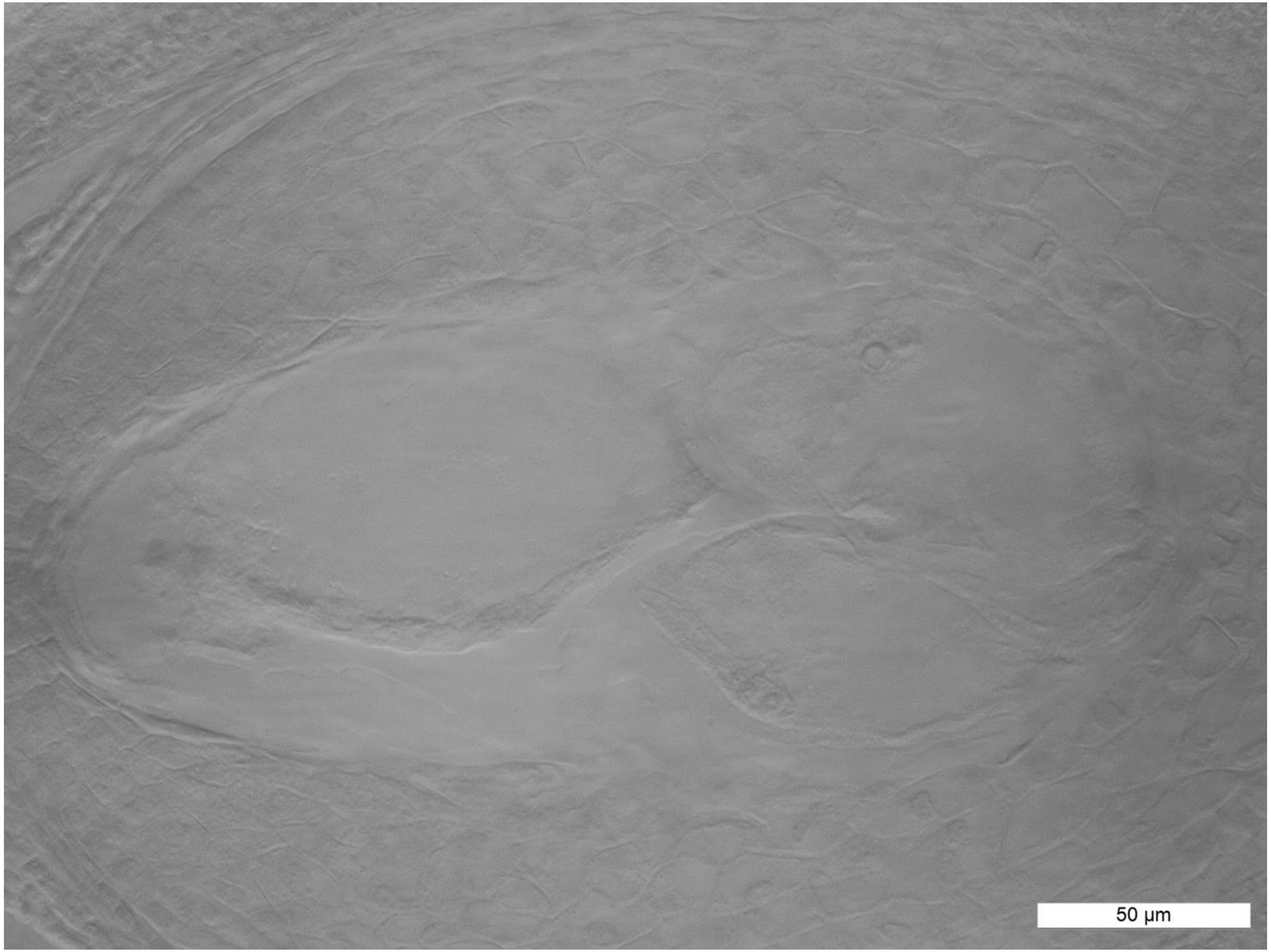

Fig. 2F ovule, antisense plant E2.13, focal plane 6

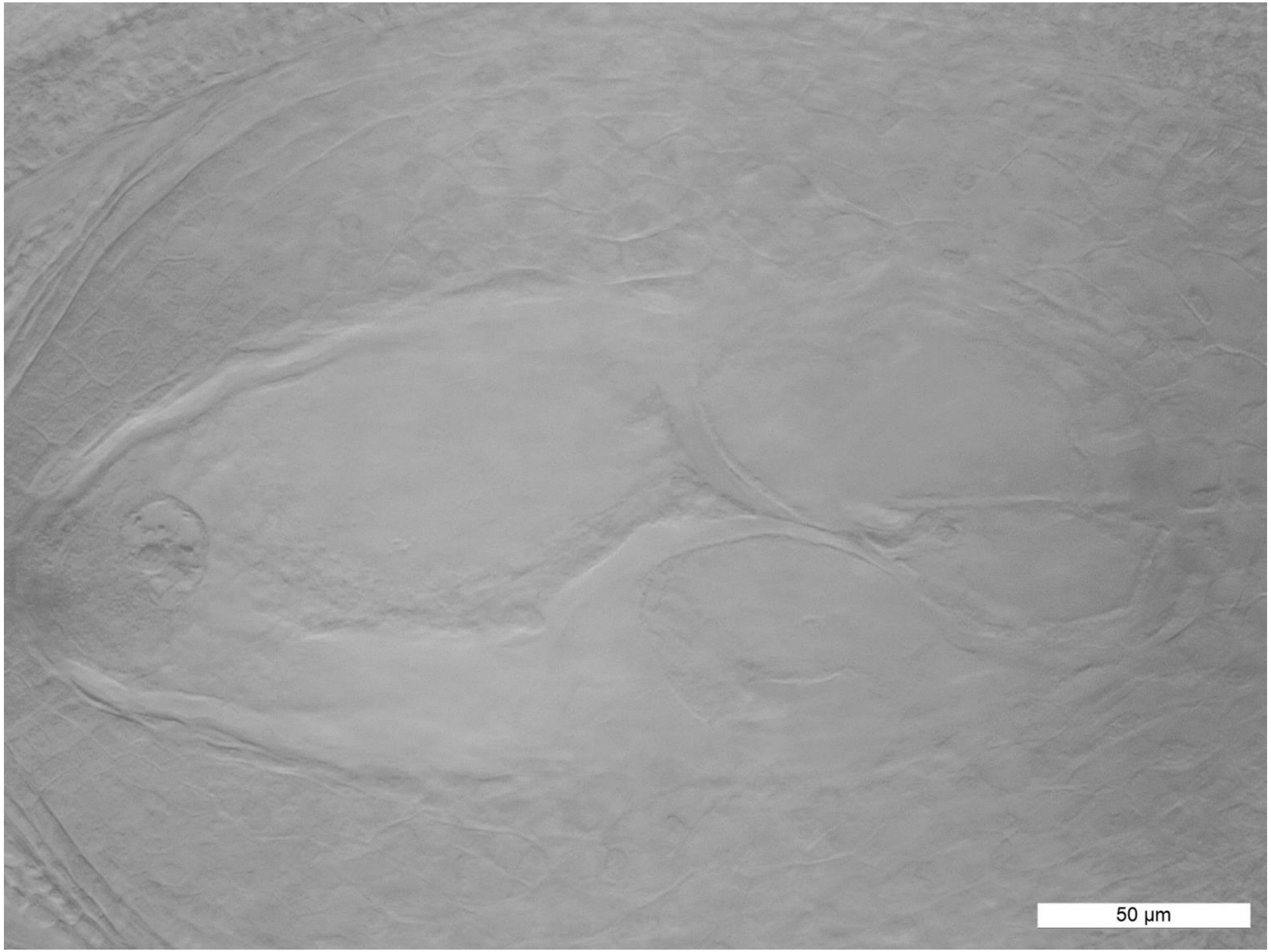

Fig. 2F ovule, antisense plant E2.13, focal plane 7

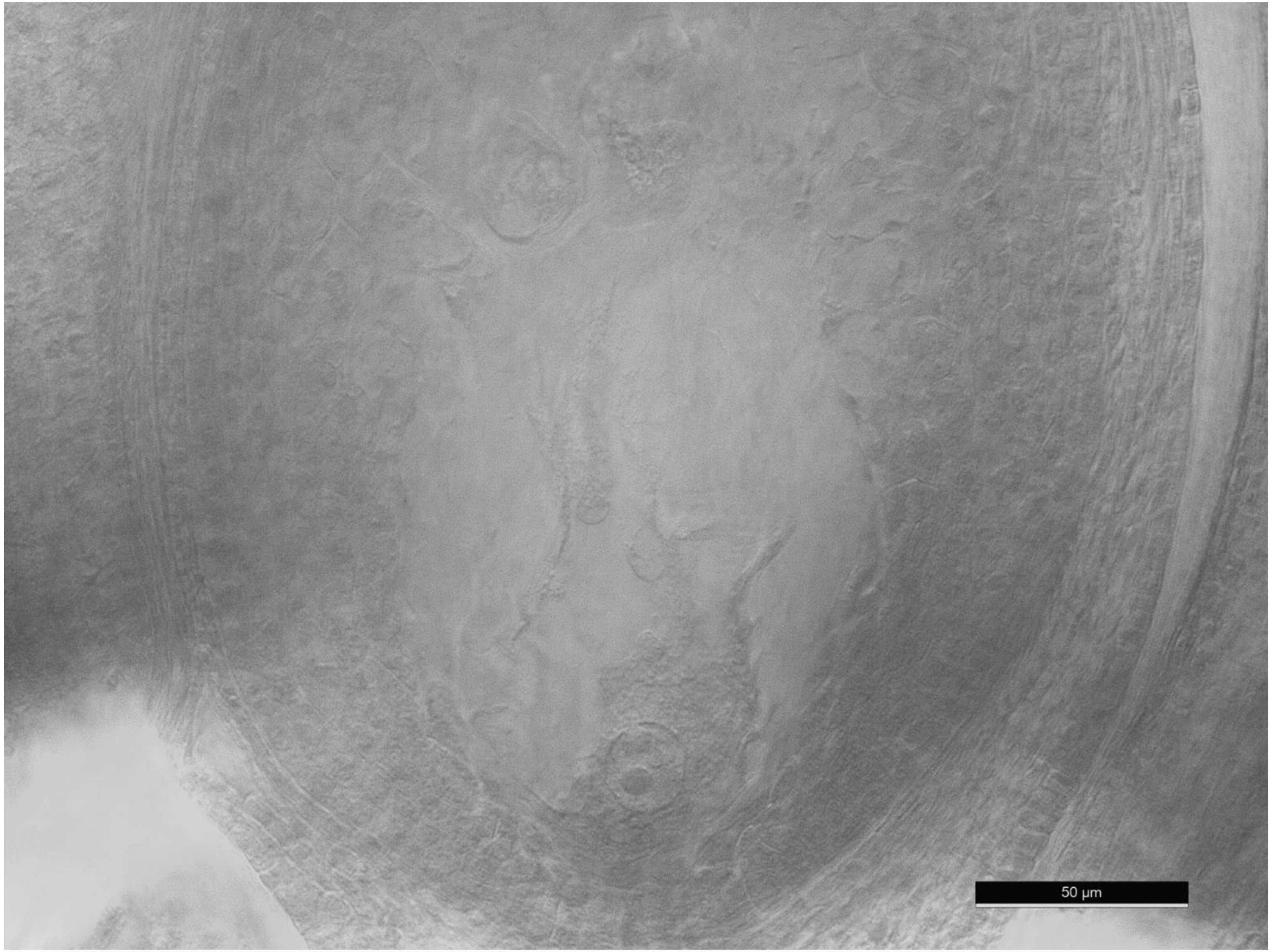

Meiotic ovule containing a single sexual embryo sac (antipodal cells at the top). Image originated from sexual control plant F58 (sexual polycross used as explant in transformation experiments). Focal plane 1.

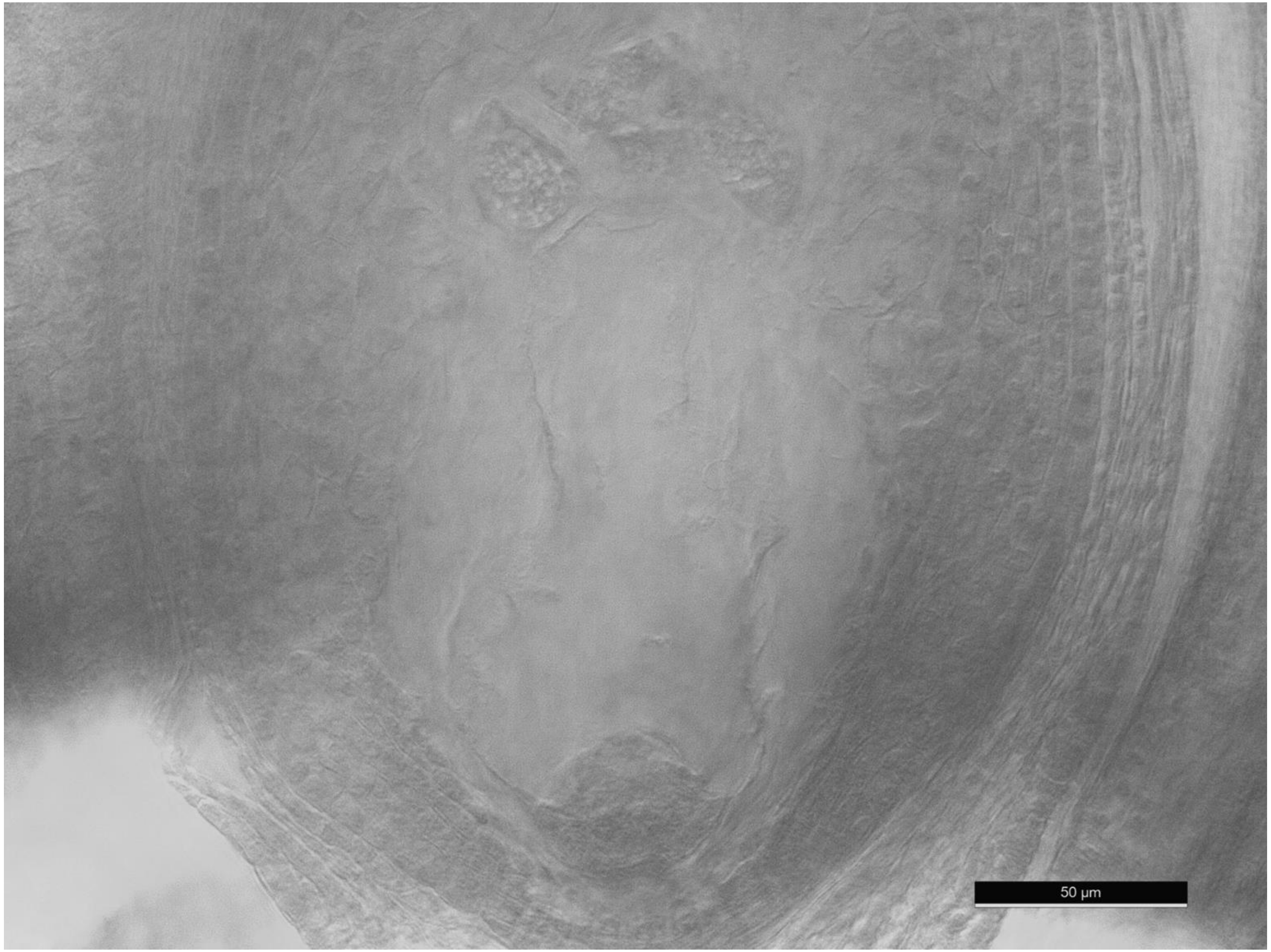

Meiotic ovule containing a single sexual embryo sac (antipodal cells at the top). Image originated from sexual control plant F58 (sexual polycross used as explant in transformation experiments). Focal plane 2.

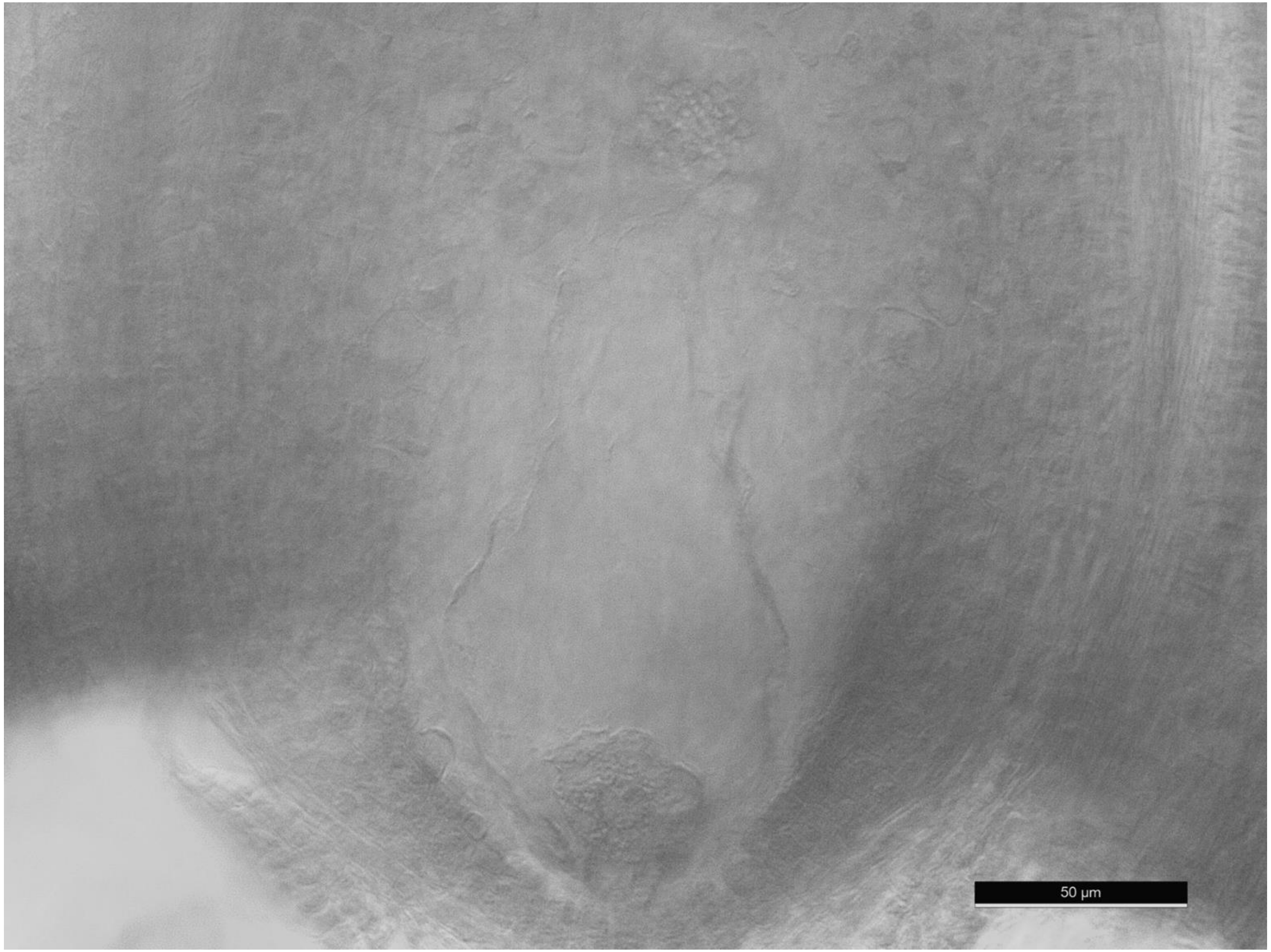

Meiotic ovule containing a single sexual embryo sac (antipodal cells at the top). Image originated from sexual control plant F58 (sexual polycross used as explant in transformation experiments). Focal plane 3.

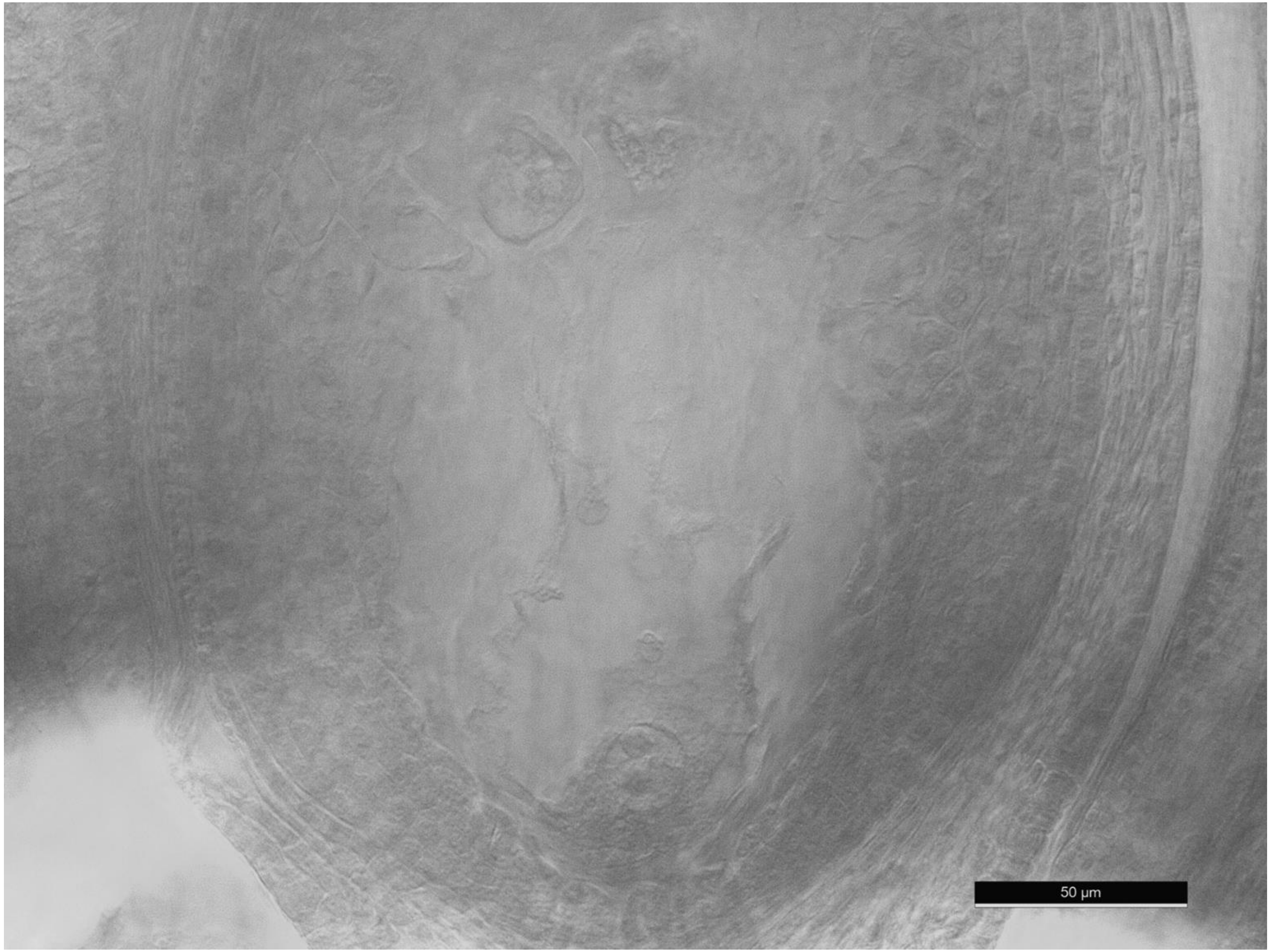

Meiotic ovule containing a single sexual embryo sac (antipodal cells at the top). Image originated from sexual control plant F58 (sexual polycross used as explant in transformation experiments). Focal plane 4.

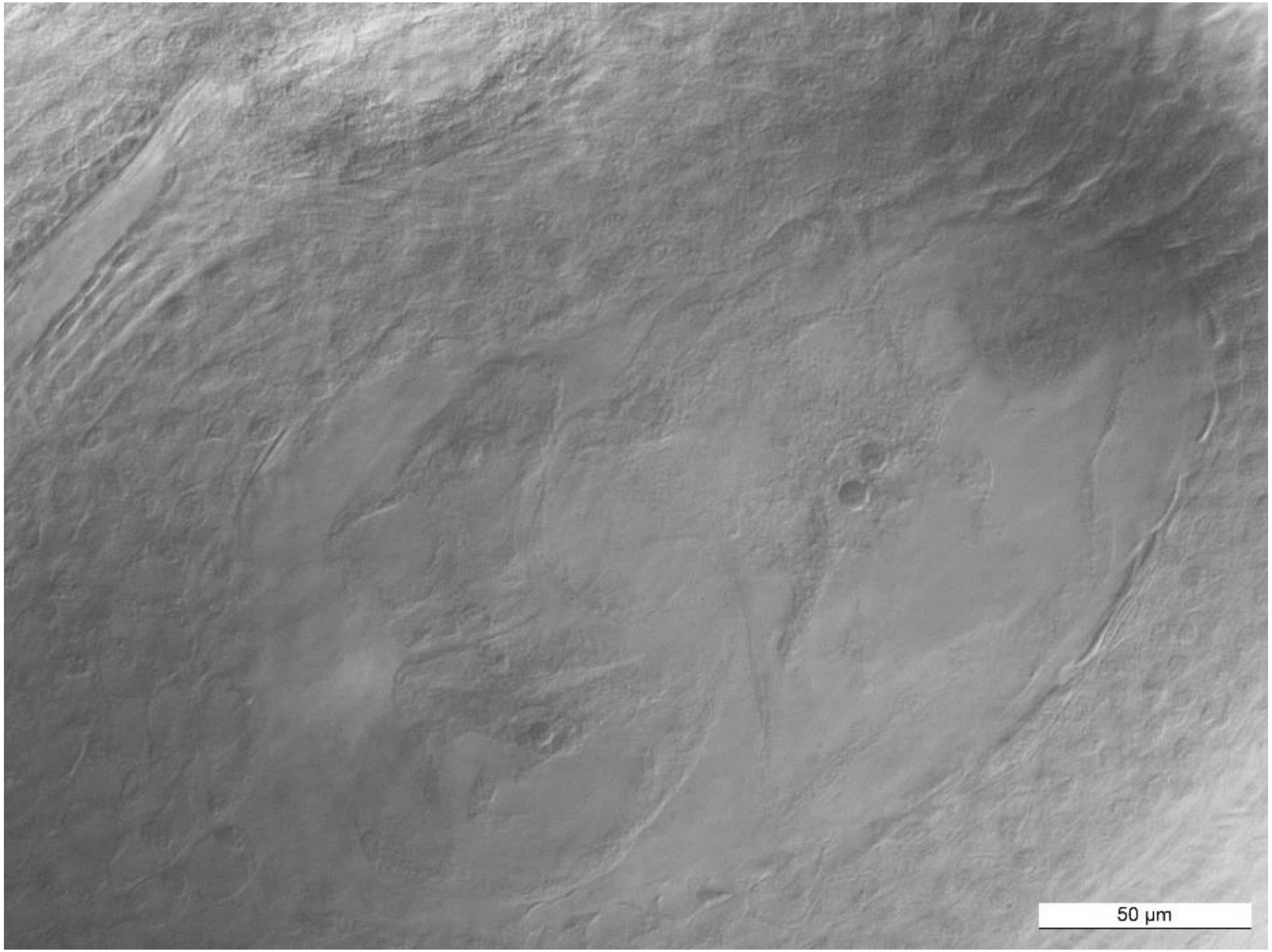

Aposporous ovule containing several mature AES, originated from obligate apomictic plant Q4117 (apomictic control), focal plane 1.

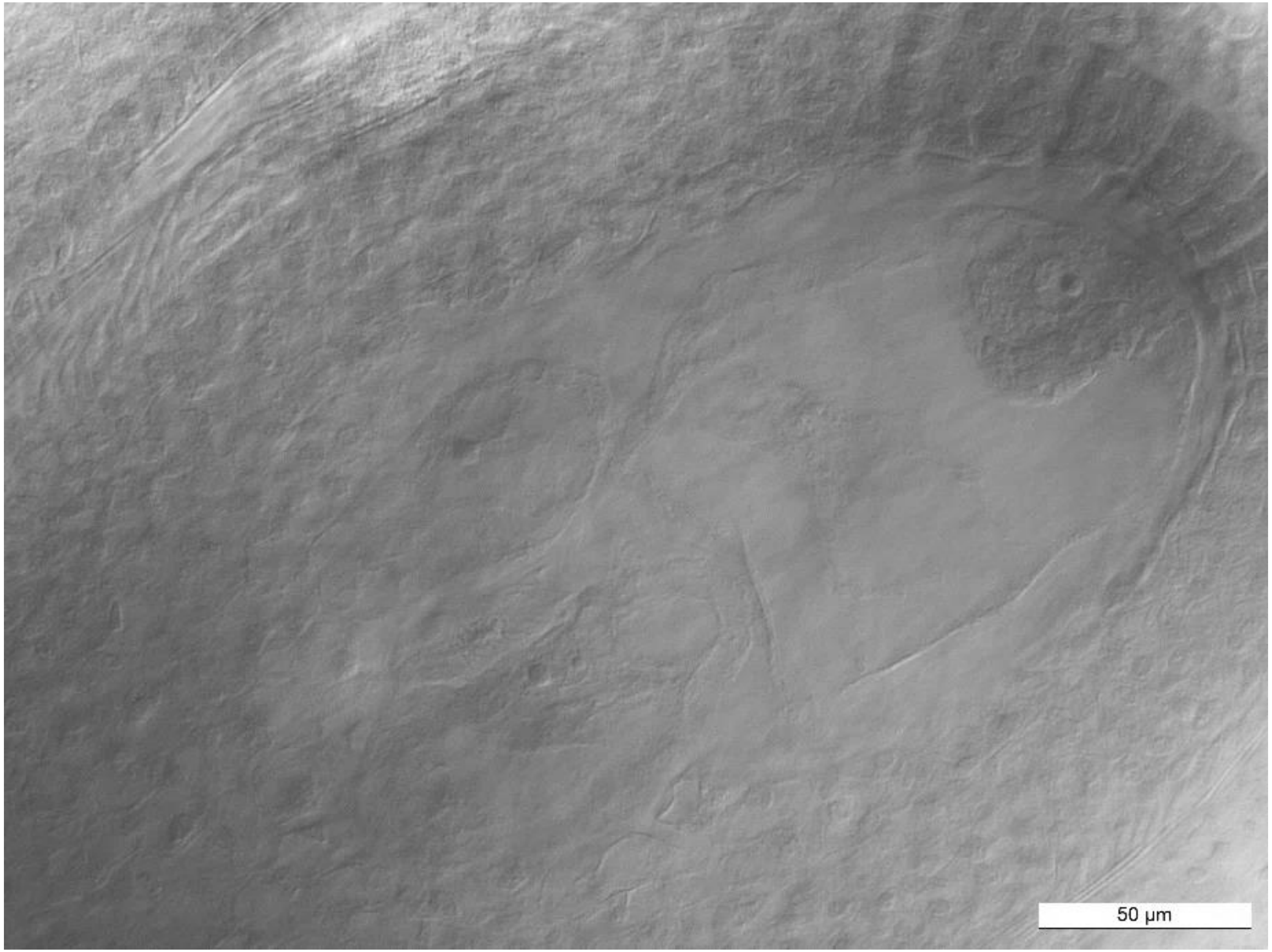

Aposporous ovule containing several mature AES, originated from obligate apomictic plant Q4117 (apomictic control), focal plane 2.

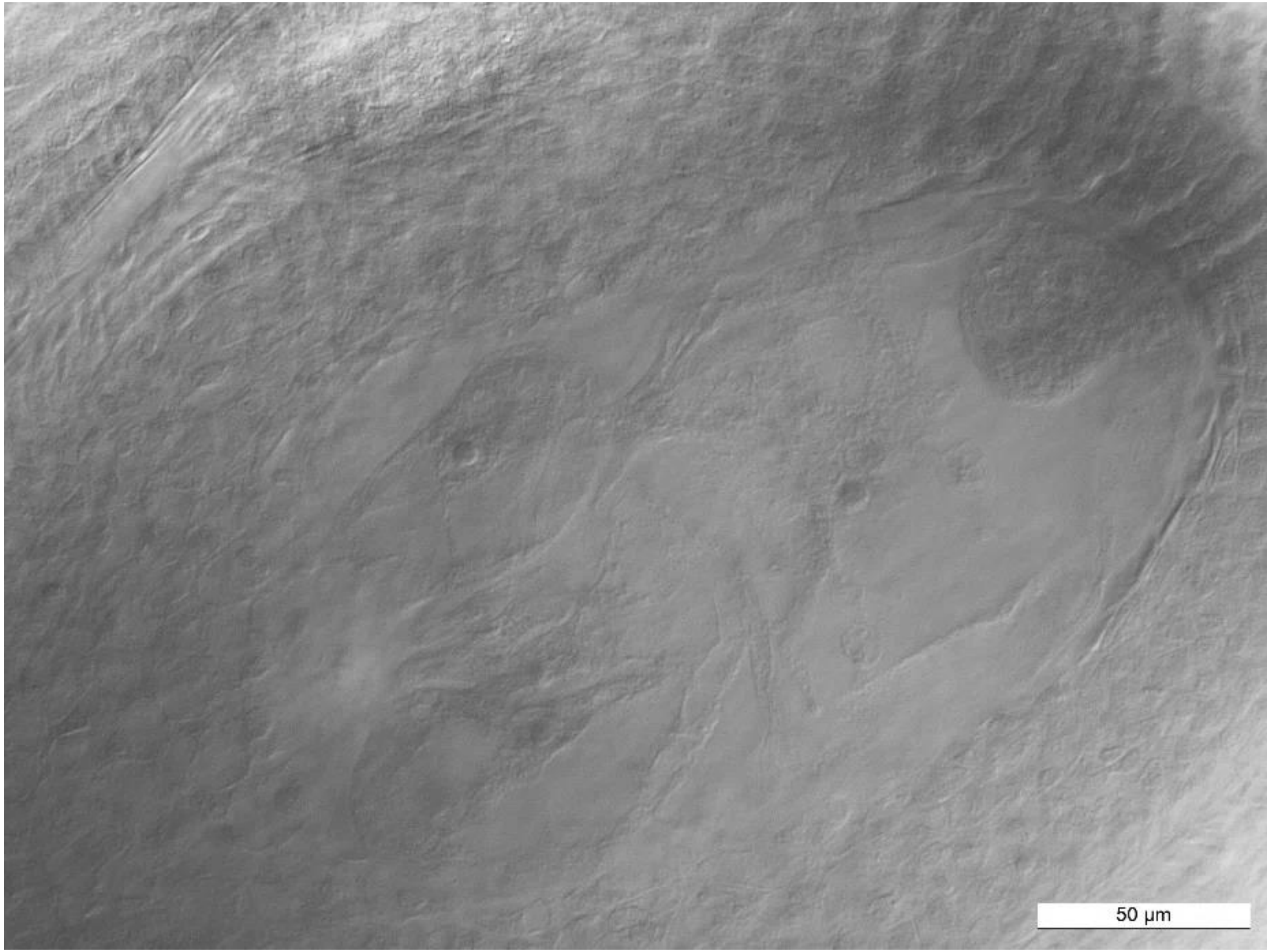

Aposporous ovule containing several mature AES, originated from obligate apomictic plant Q4117 (apomictic control), focal plane 3.

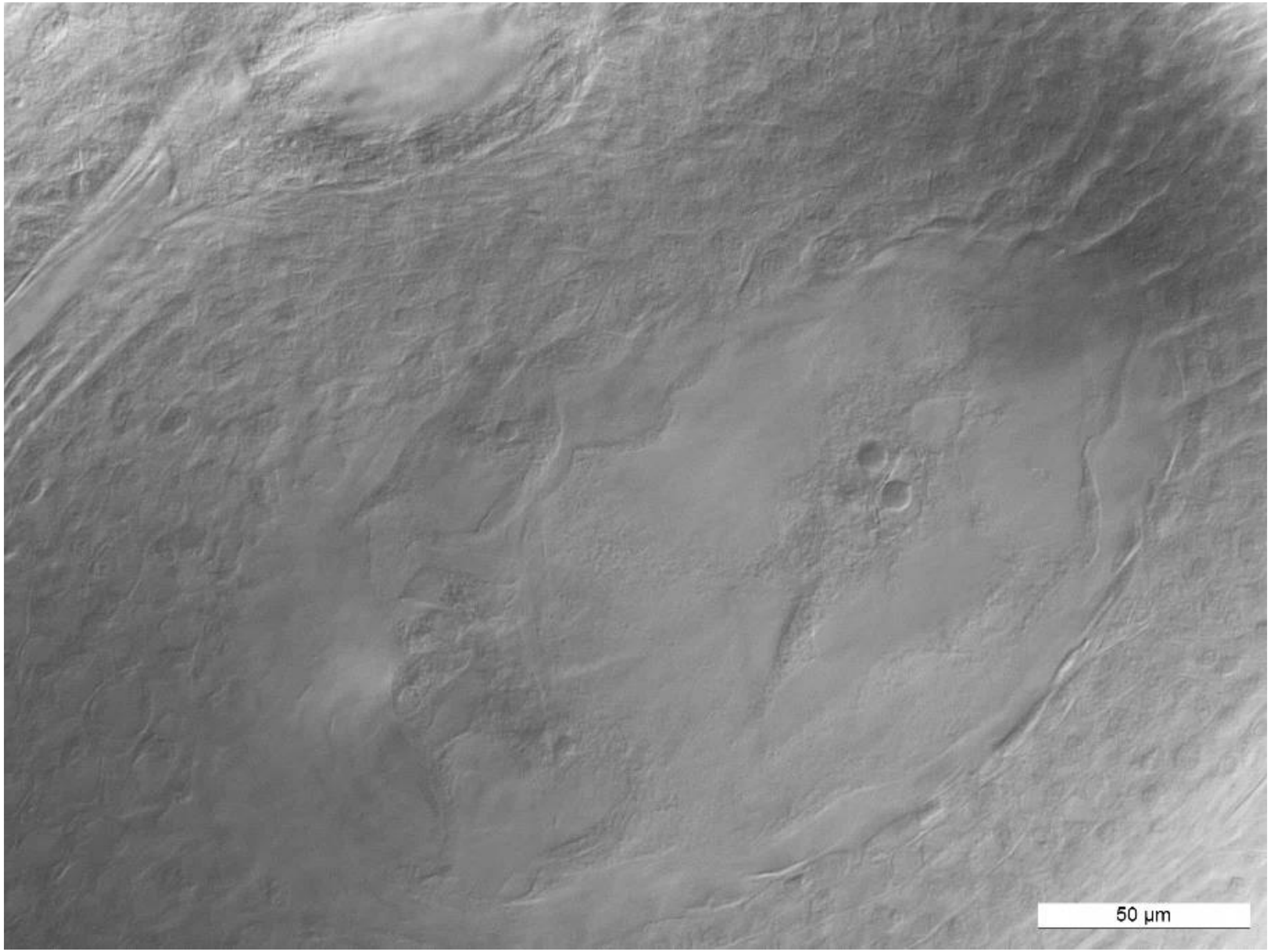

Aposporous ovule containing several mature AES, originated from obligate apomictic plant Q4117 (apomictic control), focal plane 4.
