# Supplementary figures and images for: A Plant-Specific TGS1 Homolog Influences Gametophyte Development in Sexual Tetraploid Paspalum notatum Ovules
Source: Front Plant Sci. 2019 Nov 29;10:1566. doi: 10.3389/fpls.2019.01566 (PMC6895069; doi:10.3389/fpls.2019.01566)

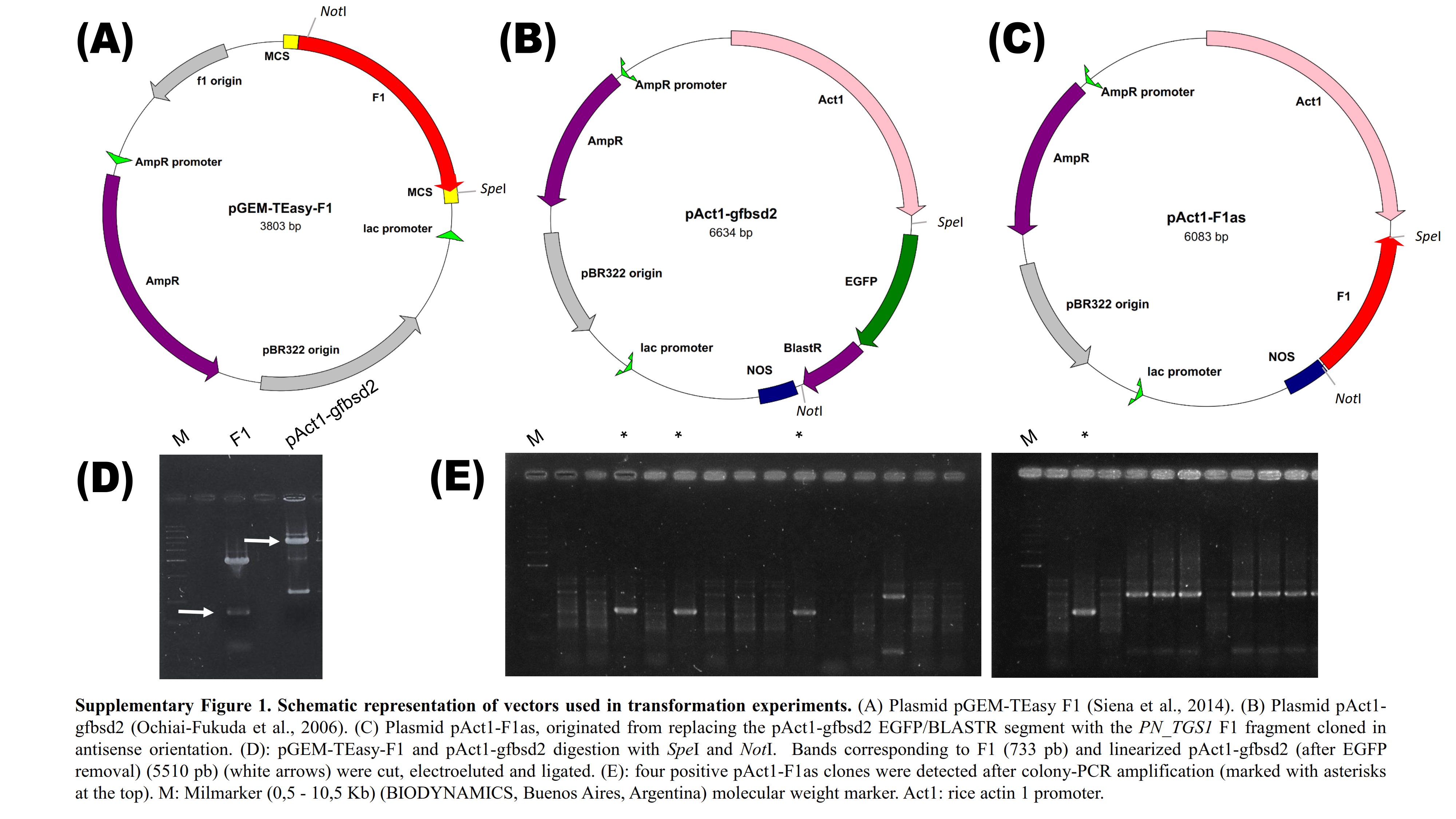

Supplement: Supplementary file 4 [file Image_1.tif]

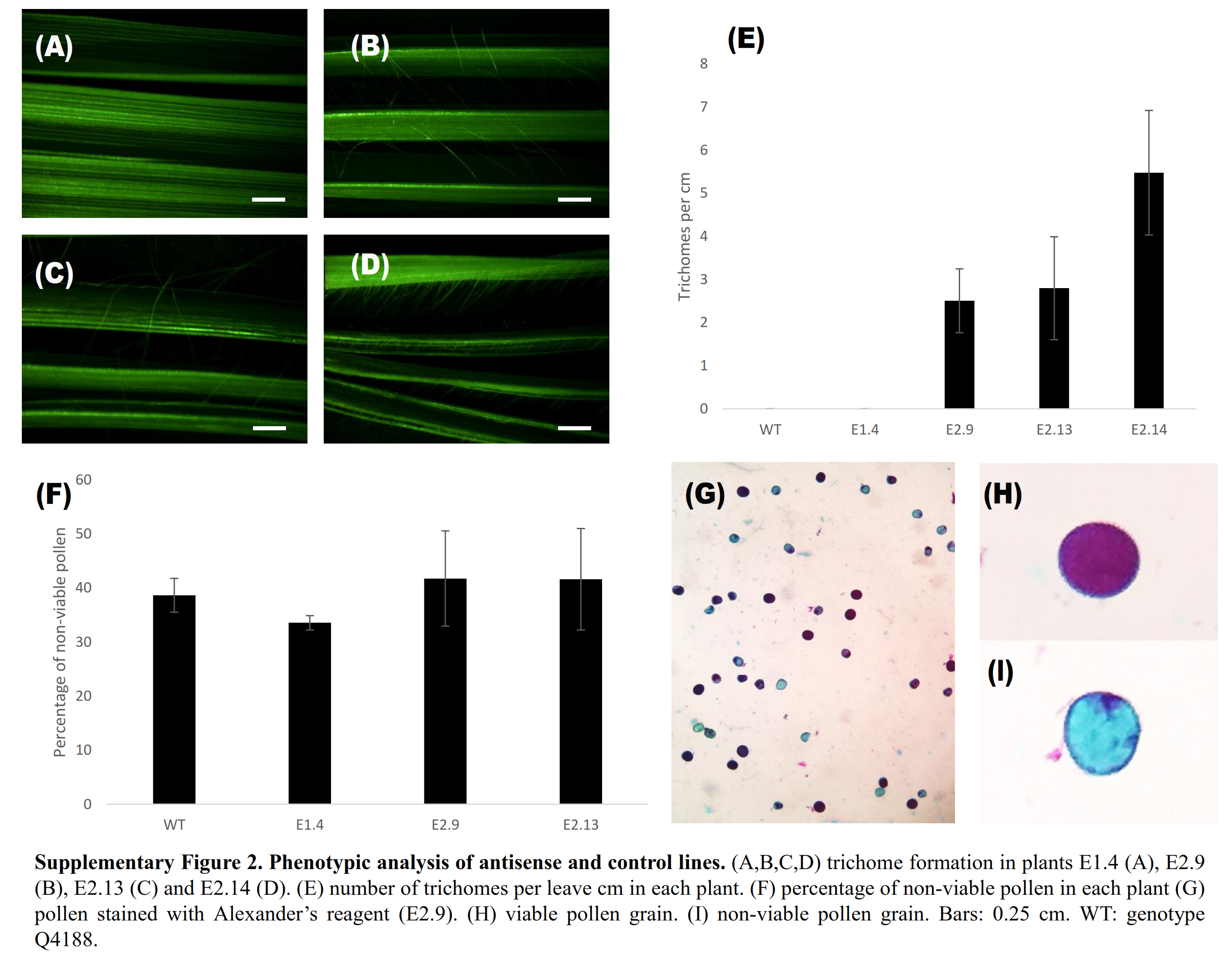

Supplement: Supplementary file 5 [file Image_2.tif]

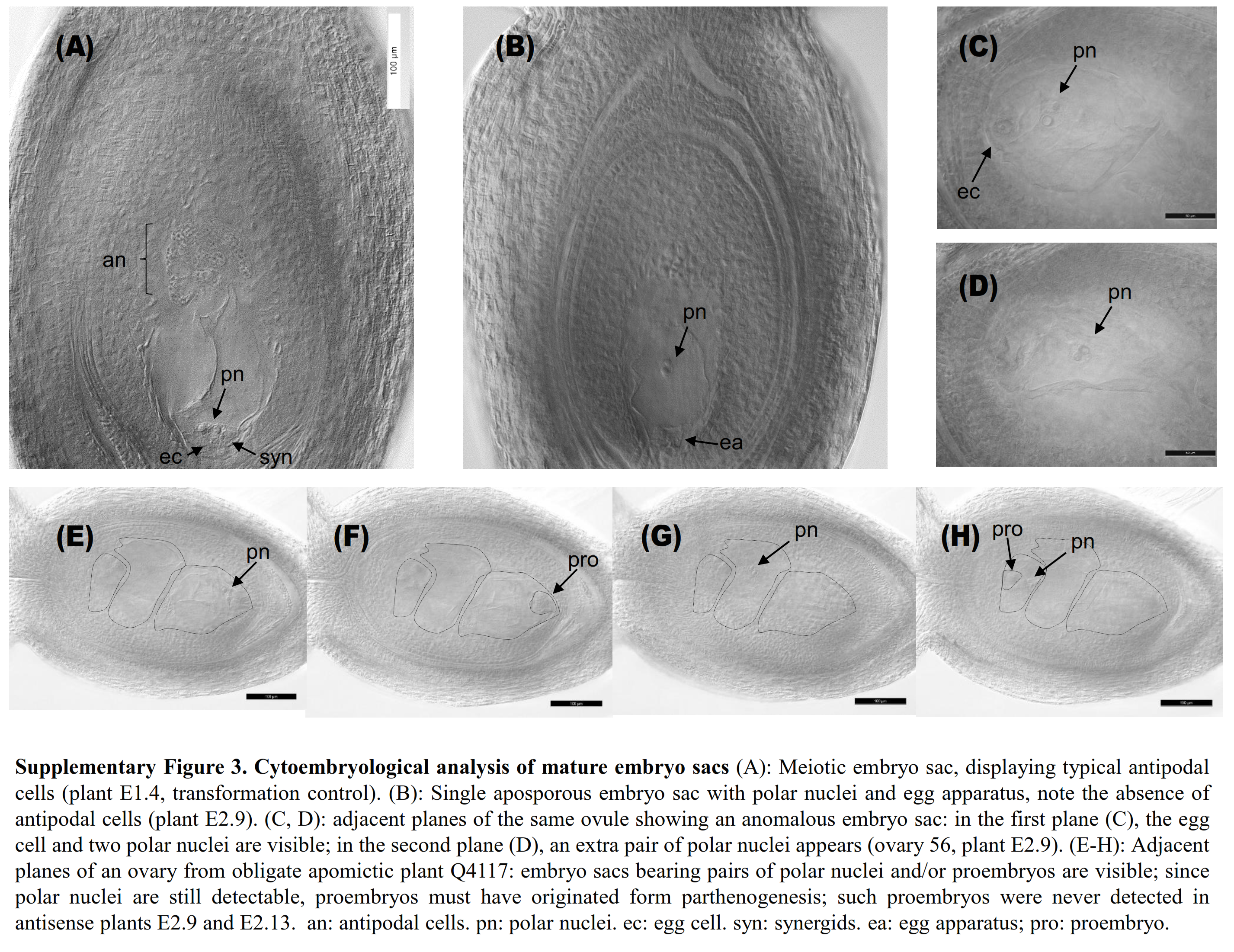

Supplement: Supplementary file 6 [file Image_3.tif]

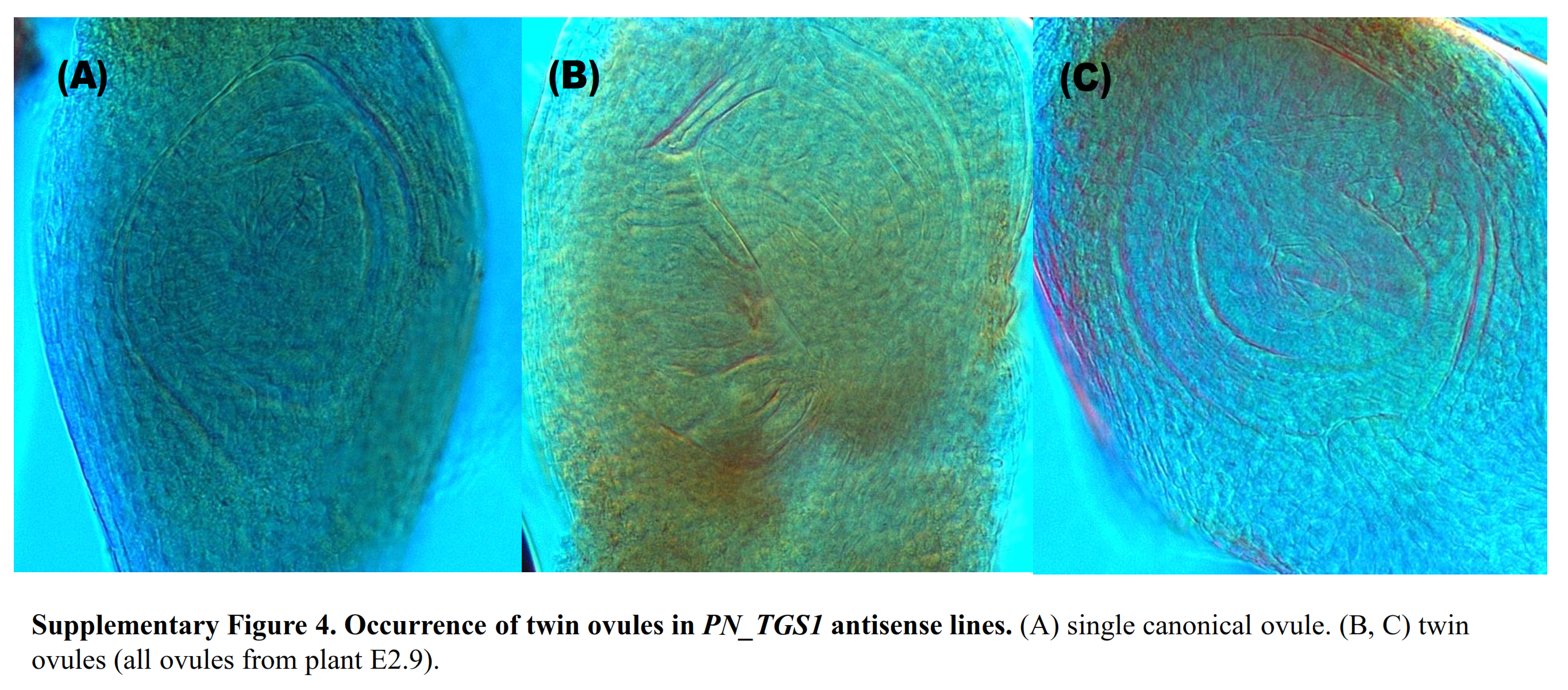

Supplement: Supplementary file 7 [file Image_4.tif]

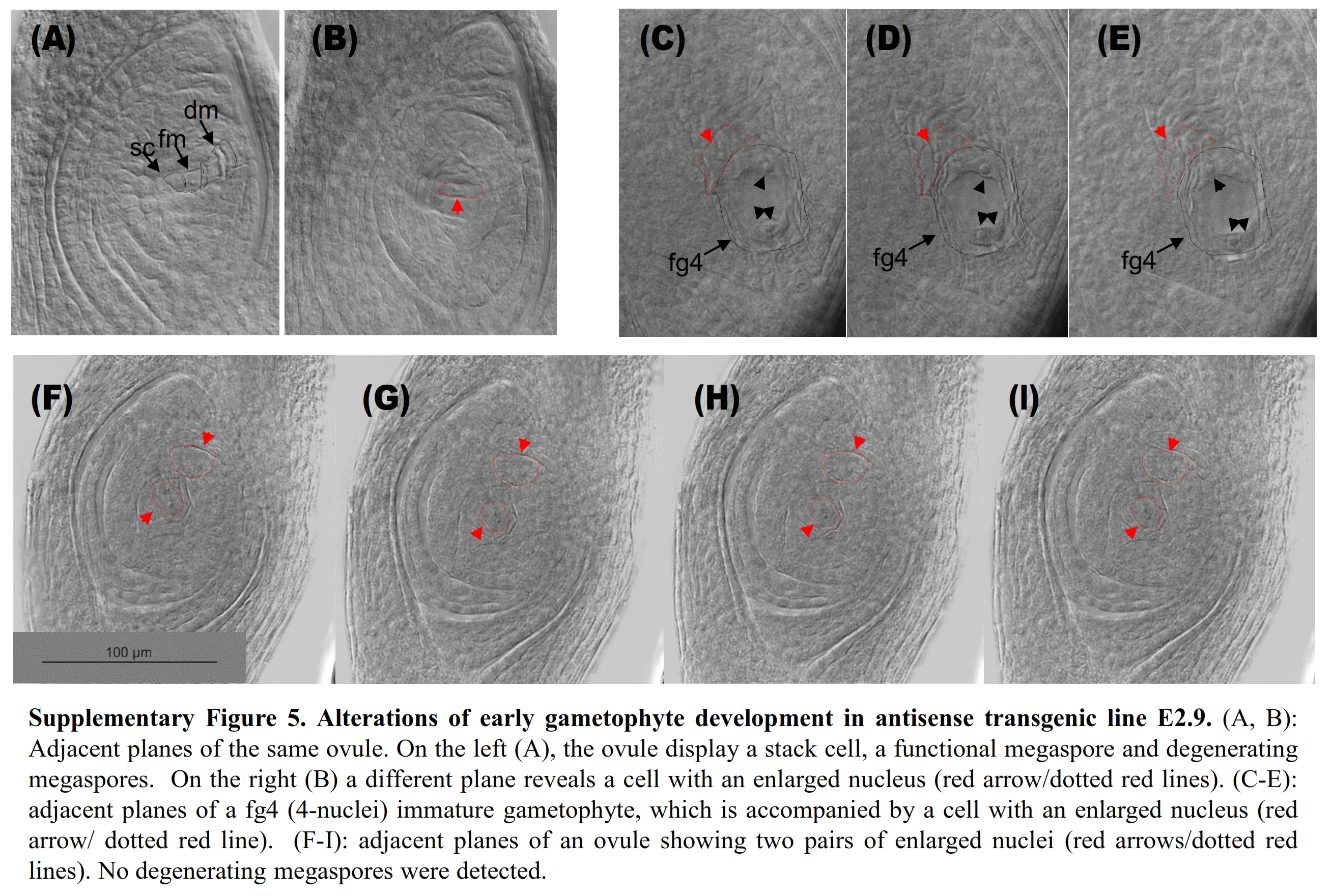

Supplement: Supplementary file 8 [file Image_5.tif]

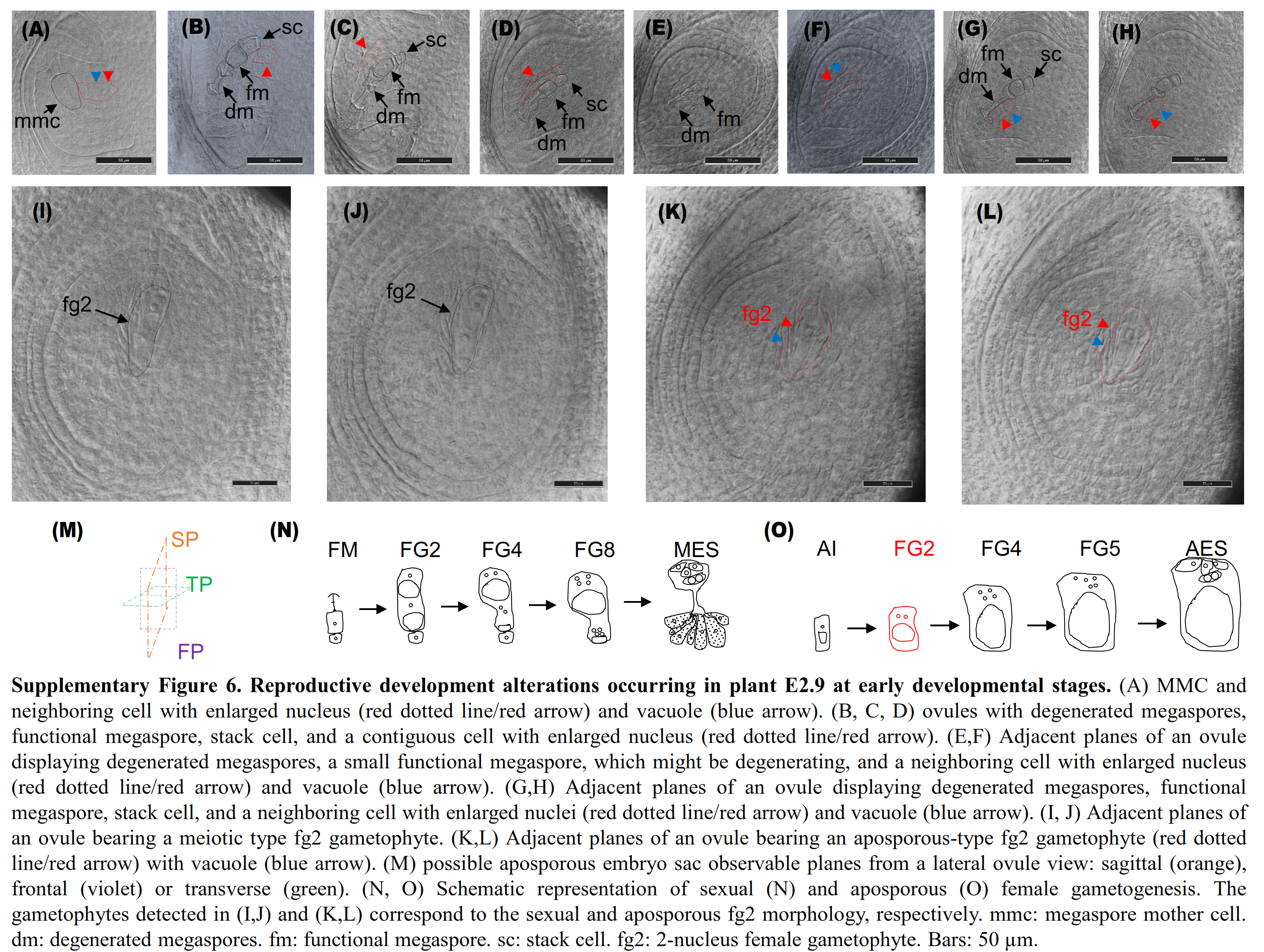

Supplement: Supplementary file 9 [file Image_6.tif]

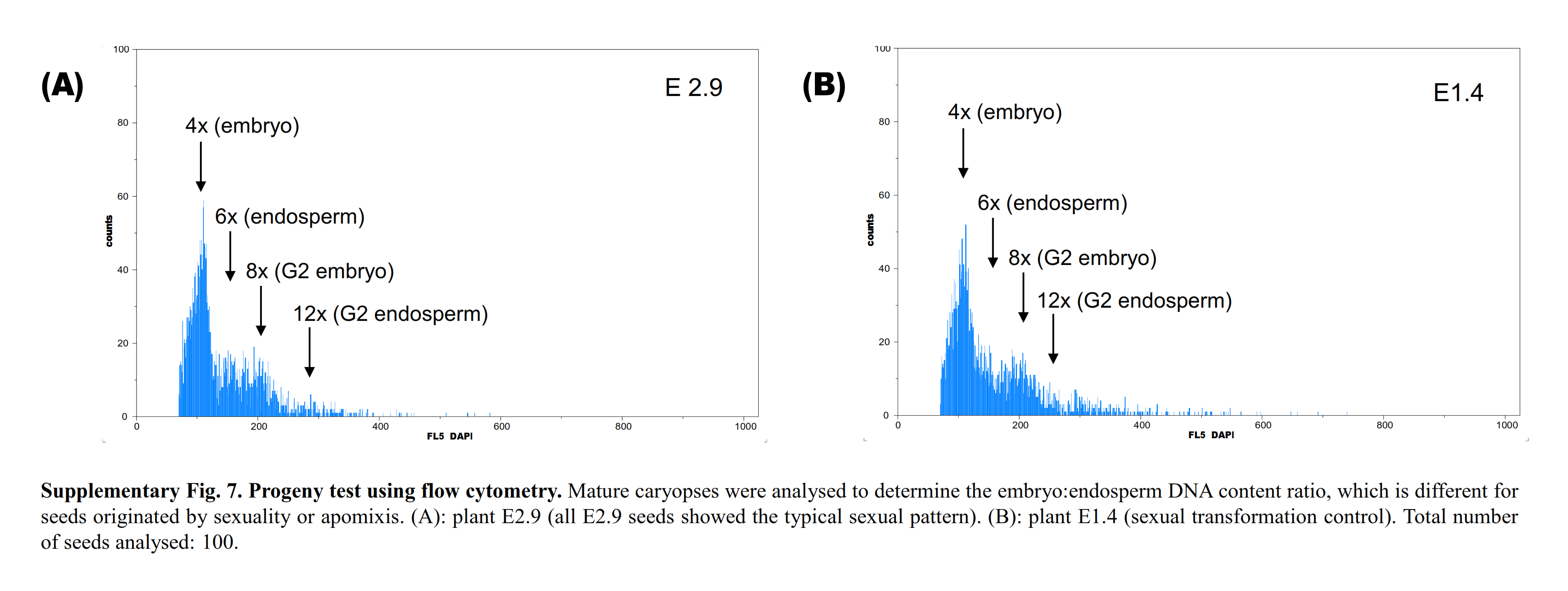

Supplement: Supplementary file 10 [file Image_7.tif]
