# Supplementary material for: A Plant-Specific TGS1 Homolog Influences Gametophyte Development in Sexual Tetraploid Paspalum notatum Ovules
Source: Front Plant Sci. 2019 Nov 29;10:1566. doi: 10.3389/fpls.2019.01566 (PMC6895069; doi:10.3389/fpls.2019.01566)
Supplement: Supplementary file 11 [file Table_1.docx]

**Supplementary Table 1: Sequence, temperature of melting/annealing and product size of the primers used in this work**

| **Primer pairs** | **Sequence** | **Tm (ºC)** | **Ta (ºC)** | **Product expected size (bp)** |
| --- | --- | --- | --- | --- |
| Act-Nos2-Upper  Act-Nos2-Lower | 5’-AAGAGGGGAAAAGGGCACTA-3’ | 60,07 | 59 | 1270 |
|  | 5’-ATTGCCAAATGTTTGAACGA-3’ | 59,02 |  |  |
| Act-Nos2-Upper  Nested-Lower | 5’-AAGAGGGGAAAAGGGCACTA-3’ | 60,07 | 59 | 530 |
|  | 5’-CAAGAACAGCCTCGCTGTAG-3’ | 59,00 |  |  |
| NOS-Upper  NOS-Lower | 5’-GGTACCGAGCTCCGAATTTCC-3’ | 60,96 | 57 | 187 |
|  | 5’-TTGCGGGACTCTAATCATAAAAA-3’ | 59,98 |  |  |
| qPCR-Upper  qPCR-Lower | 5’-ACGTTCTTGTGTTGGCATTT-3’ | 59,00 | 57 | 180 |
|  | 5’-TGCTCATCCACAGAAGATGG-3’ | 59,00 |  |  |
| ß-tubulin-Upper  ß-tubulin-Lower | 5’-GTGGAGTGGATCCCCAACAA-3’ | 63.23 | 57 | 158 |
|  | 5’-AAAGCCTTCCTCCTGAACATGG-3’ | 65.85 |  |  |
| PPIAPO-Upper  PPIAPO-Lower | 5’-CCGGTCGCAGTACAACAGAA -3’ | 62.00 | 59 | 570 |
|  | 5’-TTTCTGGACAGATCACGATCC -3’ | 62.00 |  |  |
